# Methodological quality of machine learning-based quantitative imaging analysis studies in esophageal cancer: a systematic review of clinical outcome prediction after concurrent chemoradiotherapy

**DOI:** 10.1007/s00259-021-05658-9

**Published:** 2021-12-23

**Authors:** Zhenwei Shi, Zhen Zhang, Zaiyi Liu, Lujun Zhao, Zhaoxiang Ye, Andre Dekker, Leonard Wee

**Affiliations:** 1 Department of Radiology, Guangdong Provincial People’s Hospital, Guangdong Academy of Medical Sciences, Guangzhou, China; 2 Guangdong Cardiovascular Institute, Guangzhou, China; 3 Guangdong Provincial Key Laboratory of Artificial Intelligence in Medical Image Analysis and Application, Guangzhou, China; 4 Department of Radiation Oncology (MAASTRO), GROW School for Oncology and Developmental Biology, Maastricht University Medical Centre+, Maastricht, The Netherlands; 5 Department of Radiation Oncology, Tianjin Medical University Cancer Institute and Hospital, National Clinical Research Center for Cancer, Key Laboratory of Cancer Prevention and Therapy, Tianjin’s Clinical Research Center for Cancer, Tianjin, China; 6 Department of Radiology, Tianjin Medical University Cancer Institute and Hospital, National Clinical Research Center for Cancer, Key Laboratory of Cancer Prevention and Therapy, Tianjin’s Clinical Research Center for Cancer, Tianjin, China

**Keywords:** Quantitative imaging analysis, Radiomics, Esophageal cancer, Concurrent chemoradiotherapy, Clinical outcomes, Methodological assessment

## Abstract

**Purpose:**

Studies based on machine learning-based quantitative imaging techniques have gained much interest in cancer research. The aim of this review is to critically appraise the existing machine learning-based quantitative imaging analysis studies predicting outcomes of esophageal cancer after concurrent chemoradiotherapy in accordance with PRISMA guidelines.

**Methods:**

A systematic review was conducted in accordance with PRISMA guidelines. The citation search was performed via PubMed and Embase Ovid databases for literature published before April 2021. From each full-text article, study characteristics and model information were summarized. We proposed an appraisal matrix with 13 items to assess the methodological quality of each study based on recommended best-practices pertaining to quality.

**Results:**

Out of 244 identified records, 37 studies met the inclusion criteria. Study endpoints included prognosis, treatment response, and toxicity after concurrent chemoradiotherapy with reported discrimination metrics in validation datasets between 0.6 and 0.9, with wide variation in quality. A total of 30 studies published within the last 5 years were evaluated for methodological quality and we found 11 studies with at least 6 “good” item ratings.

**Conclusion:**

A substantial number of studies lacked prospective registration, external validation, model calibration, and support for use in clinic. To further improve the predictive power of machine learning-based models and translate into real clinical applications in cancer research, appropriate methodologies, prospective registration, and multi-institution validation are recommended.

**Supplementary Information:**

The online version contains supplementary material available at 10.1007/s00259-021-05658-9.

## Introduction

Esophageal cancer (EC) is the seventh most common malignancy, and the sixth most common cause of cancer-related death worldwide [1]. Prognosis for EC patients remains poor to date, with a 5-year survival chance of 20% [2]. Although the histopathology and disease characteristics differ between eastern and western countries due to genetic variations, concurrent chemoradiotherapy (CCRT) plays an important global role in the treatment of EC [3].

The CROSS trial was a landmark study that established the role of neoadjuvant chemoradiotherapy (nCRT), and laid the foundation of nCRT as the standard of care for *resectable* EC [4]. While CROSS demonstrated that nCRT improved average survival among EC patients and side-effect rates were acceptable, it remains clinically meaningful to select patients that will personally benefit from nCRT versus their probable side effects. Definitive chemoradiotherapy is the standard of care for *unresectable* EC [5]. However, it remains difficult to predict individual outcomes (e.g., treatment response) of any type of CCRT due to tumor heterogeneity between subjects and complex tumor microenvironments within.

Technical advances in radiation delivery such as modulated radiotherapy, image guidance, and scanning proton beams have vastly improved target coverage and avoidance of adjacent healthy organs. It is practically impossible to entirely avoid some unintended damage to nearby organs, which results in radiotherapy complications. A way to predict treatment response and side effects at the earliest step of CCRT works hand in hand with radiotherapy technology and new drug therapies, and this is essential to guide individually personalized treatment, to improve the survival likelihood and to retain high quality of remaining life for EC patients.

The spatial and time heterogeneity of solid tumors at the genetic, protein, cellular, microenvironmental, tissue, and organ levels makes it difficult to accurately and representatively characterize a tumor using only invasive sampling methods, such as pathology and molecular imaging examination. Quantitative analysis based on volumetric non-invasive imaging (i.e., radiomics [6–8]) suggests the attractive hypothesis of measuring whole-tumor heterogeneity in vivo. Radiomics makes it feasible to characterize whole-tumor heterogeneity and also monitor tumor evolution over time.

Radiomics requires large volumes of clinical imaging data to be converted into a vast number of numerical features with the assistance of computers, which can then be mined for clinically actionable insights using high-dimensionality machine learning methods. Radiomics includes features that are defined a priori by human operators (i.e., “handcrafted” features) as well as purely data-driven features arising via end-to-end training of deep learning neural networks. A number of key studies and evidence syntheses have shown that radiomics has potential to recognize heterogeneity in primary tumors and/or lymph nodes in a variety of cancers with clinical applications for diagnosis and prognostication [9–12].

Within EC, radiomics is presently an active area of original research (e.g., in [13, 14]), but at time of writing, there has been no comprehensive PRISMA-compliant (Preferred Reporting Items for Systematic Reviews and Meta-Analyses) systematic review of radiomics specifically addressing *methodological robustness and clinical relevance* of radiomics for patients with EC treated by CCRT. In this systematic review, we present to the reader a cohesive critical appraisal of research up to date, and a summary of clinical relevance of radiomics as a potential tool for predicting (i) treatment outcomes, (ii) longer-term prognosis, and (iii) CCRT treatment-related toxicity.

## Materials and methods

### Eligibility criteria

We conducted this systematic review from May to June 2021, in accordance with PRISMA guidelines [15]. In this study, we included only primary observational studies published between May 2011 and June 2021 using either handcrafted and/or deep learning-based radiomics features extracted from clinical imaging—specifically computed tomography (CT), magnetic resonance (MR), and positron-emission tomography (PET)—to develop clinical prediction models on human primary EC subjects treated by CCRT. Articles eligible for critical appraisal had to be published as full texts in peer-reviewed journals in the English language within the last 5 years.

### Exclusion criteria

Diagnostic accuracy studies evaluating tumor differentiation grade or the diagnosis of lesions were excluded. Studies that exclusively addressed modelling on non-radiomic features, such as only standardized uptake value (SUV), clinical parameters, and/or dosimetric parameters, were excluded. Clinical outcomes that were primarily associated with surgery alone, radiotherapy alone, or chemotherapy alone were excluded. Case reports, other (systematic) reviews, conference abstracts, editorials, and expert opinion papers were also excluded.

### Search methods

An initial citation screening in PubMed and EMBASE electronic databases was performed on 9 May 2021. We used a search string containing Medical Subject Headings (MeSH) or Emtree terms for “esophageal cancer” combined with other text words that related to outcomes, prediction, model, radiomics (including textural analyses and quantitative analyses), and artificial intelligence. The search filters used are provided in the Supplementary Material Table S1. Articles were also included for screening based on prior knowledge of the authors. We searched the reference section of reviewed papers for any additional articles that may have been missed in the electronic databases.

### Selection process

Two authors (Z.Z. and L.W.) worked independently on screening PubMed and Embase records, based on titles and abstracts alone. Candidate articles were combined, and then, any disagreements were resolved by consensus; a third author (Z.S.) was available for adjudication but was not required. Full text of the candidate articles was obtained using an institutional journal subscription, and examined in detail for eligibility against the aforementioned criteria. Only full-text articles unanimously deemed eligible for review were then included for detailed data extraction and critical appraisal.

### Data extraction

Two authors (Z.S. and Z.Z.) independently performed extraction of publication details and clinical outcomes. From the eligible articles, information pertaining to general study characteristics were extracted (author, publication year, primary cancer type, imaging protocol, treatment modality, sample size) together with radiomics feature-related descriptions (deep learning-based or/and handcrafted features, software used for feature extraction, and whether radiomics features were combined with non-radiomics predictors). Model characteristics and primary reported findings of the included studies were also extracted and summarized, which included use of retrospectively/prospectively collected patient personal data, the collaborating institution(s), sample sizes used to build the model, number of radiomics features initially considered versus that retained in the final model, type of model assessed, the reported performance metrics, and results of model calibration if given.

### Methodological robustness

Classical evaluation tools such as Quality in Prognostic Studies (QUIPS) for prognostic studies [16], Quality Assessment of Diagnostic Accuracy Studies-2 (QUADAS-2) for diagnostic tests [17], and Prediction model Risk Of Bias ASsessment Tool (PROBAST) [18] were not specifically designed for high-dimensional predictive modelling studies such as radiomics. Lambin et al. [19] proposed a radiomics quality score (RQS) that assigned “points” to various steps in radiomics modelling workflow, and such RQS evaluation approach has been previously used [20–24] in reviews. However, specialist evidence synthesis communities (such as the Cochrane Collaboration) advise that a single numerical score may not be appropriate to capture a complex question such as overall methodological robustness of a diagnostic/prognostic model. Other reviewers have also used Transparent Reporting of a multivariable prediction model for Individual Prognosis Or Diagnosis (TRIPOD) [25] type as a surrogate measure for quality, but it must be re-emphasized that TRIPOD is a model reporting guideline, not in fact a critical appraisal checklist.

In this work, we have applied an assessment metric guided by the RQS together with findings of other radiomics methodological evaluations [26, 27]. Due to the rapid changes in machine learning and radiomics expertise in the relevant scientific community, we limited the methodological quality appraisal to the included studies published within the past 5 years. The appraisal was initially performed independently by two authors (Z.S. and Z.Z.) and then combined. Disagreements were resolved by consensus, and an experienced senior author (L.W.) adjudicated on differences of evaluation. Each methodological criterion was provided a consensus rating of “good,” “moderate,” or “poor,” based on 13 specific quality criteria:

1. It would have been ideal if a *detailed study protocol with its statistical analysis plan had been prospectively registered in an open access registry prior to commencement*. Studies that used prospectively collected patient data was rated as “moderate” since the study plan would probably have been registered during internal ethical review. Absence of any of the above was deemed “poor.”
2. For reproducibility and comparison between institutions, it is important to provide *detailed information that documents the image acquisition conditions*. Typical information might include scanner make/model, scan protocol, enhanced/unenhanced CT scans, tube voltage, tube current, slice thickness, and voxel size appropriate to the imaging modality examined. Partial or incomplete information was rated “moderate,” but its absence in text or supplemental was deemed “poor.”
3. It is widely known that digital image preprocessing steps can strongly influence the quantitative image analysis results that follow. Studies that give *detailed information to reproduce the pre-processing steps* (typically includes filters for de-noising, intensity normalization, and voxel resampling). Partial or incomplete information was rated “moderate,” but its absence in text or supplemental was deemed “poor.”
4. The *method by which the region of interest (ROI) for analysis has been defined* can also influence the generalizability of radiomics models. For instance, automated or semi-automated delineation of organs may be more consistent than manual delineation. A “good” score was given for full information on ROI delineations, including review by experienced experts and/or any inter-observer sensitivity checks. Partial information or no information were scored “moderate” and “poor,” respectively.
5. Radiomics studies typically consider a massive number of features relative to the sample size and the event rate of the outcome of interest; therefore, *feature selection/dimensionality reduction steps* are generally needed to reduce risk of overfitting. We deem that reproducibility and repeatability tests of feature stability, and/or unsupervised feature selection methods (such as principal components analysis or clustering), prior to applying supervised learning with the outcome of interest, would be “good.” Partial documentation or inadequately justified methods were deemed “moderate,” otherwise “poor” when there was a high risk of either over-fitting or false positive association.
6. *Potential correlations should be examined between radiomics and non-radiomics (other biological) features*, since this can identify possible confounders and justify the added value of imaging features. Adequate checks for possible correlations are deemed “good,” insufficient or limited checks as “moderate,” or if such checks were not attempted then “poor.”
7. Since the general idea of a prognostic model is to permit stratification of patients, it is important for studies to provide *clear justification for defining risk groups*, including how risk thresholds and optimum operating points had been determined. Stratification based on clinical argumentation or agnostically using median or standard cutoffs (e.g., class probability of 0.5) was deemed “good.” Use of optimally “tuned” cutoffs or deriving risk groups as part of the model optimization step can introduce some loss of robustness, and were thus deemed “moderate.” No justification or lack of documentation in this regard was scored as “poor.”
8. As emphasized by TRIPOD, *model performance should be evaluated with an external validation cohort*, ideally with fully independent researchers, scanners, delineations, etc. Model performance metrics with strong support in external validation (TRIPOD type III) would have been rated as “good.” Validation by non-random split from the training cohort (e.g., by time, location, or some other pre-treatment characteristic) or by multiple repeated random sampling (k-folds, bootstrapping) were rated “moderate.” However, one-time random sampling or no report of model validation at all was rated as “poor.”
9. Models utilizing radiomics features should be able to show *added value when compared against, or combined with, clinical and/or non-radiomics models*. We defined the presence of sufficient description about comparison with clinical/non-radiomics model or holistic models as “good,” inadequate comparison as “moderate,” and otherwise as “poor.”
10. *Model performance should be reported in terms of appropriate discrimination metrics*, such as c-index for time-to-event models and AUC for binary classification models. A study was deemed “good” if it reported discrimination metrics for training and test dataset (or other related metrics) together with confidence intervals and statistical significance. Partial information about discrimination was deemed “moderate,” or if no information was provided then “poor.”
11. As recommended in TRIPOD, *model calibration should also be reported in addition to its discriminative performance*. A “good” study provided a test of calibration or goodness-of-fit results, together with a calibration figure. Partial information about calibration was deemed “moderate,” or if no calibration results were given then “poor.”
12. For ease of implementation, studies should *discuss the potential clinical utility of their model(s) and provide some justification for use*, such decision curves analysis or cost–benefit analysis. We defined the presence of an estimated clinical utility as “good,” partial or inadequate analysis as “moderate,” and otherwise as “poor.”
13. Studies should *report parameters of their model(s) in ample detail to permit independent external validation*. Those studies rated “good” provided the reader with regression coefficients for each feature or otherwise made it possible to calculate risk scores, such as making their model(s) accessible via an online repository or by providing a calculation aid (e.g., a nomogram). Studies that only reported features selected in the final model were deemed “moderate”; however, studies that did not provide adequate information to independently validate the model were rated “poor.”

### Objectives

The primary objective was to estimate the overall ability of radiomics models, or models containing some radiomics information, to predict clinical outcomes that are of particular clinical interest in CCRT for EC. This gives us a picture of the current status of clinical readiness of radiomics as a potential tool for clinical decision-making and/or possible incorporation of radiomics-powered models into holistic decision support systems. Secondly, we included a critical appraisal of reported model performance against the methodological robustness (i.e., internal validity) because this is key for understanding its clinical applicability, and such robustness informs the degree of wide generalizability (i.e., external validity) that might be expected from a reported model.

## Results

### Literature search results

**Fig. 1:**
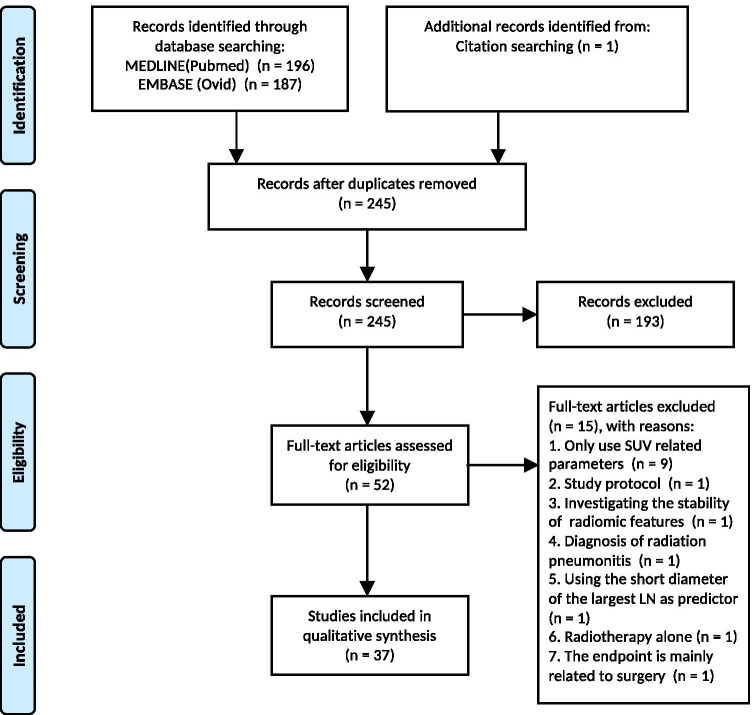
Flowchart of the literature search and study selection (PRISMA 2009 [65])

A PRISMA flowchart diagram illustrating article selection is shown in Fig. 1. A total of 384 records were identified based on the specified search terms (MEDLINE/PubMed *n* = 196, EMBASE *n* = 187, and one was found in the cited references of an included article). After duplicates removal, there were 245 articles available for screening. Applying the selection criteria led to 52 studies for full-text screening. At the end, a total of 37 articles were deemed eligible [28–64], including 30 articles within 5 years [28–38, 41–43, 45, 47–50, 52–54, 57–64].

### Overall characteristics of included studies

Table 1 and Supplementary Material Table S2 summarize the general characteristics across all included studies. The majority (20 of 37) of studies combined both esophageal squamous cell carcinoma (ESCC) and esophageal adenocarcinoma (EAD) patients. There were 13 studies conducted exclusively on ESCC patients but only two studies on EAD patients alone. Two other studies did not actually mention the histopathology type of the cohorts studied.

**Table 1:** Summary of general study characteristics

Ref	Cancer type (recruitment period)	Imaging modality	Imaging acquisition settings	Treatment	Sample size	Type of features	Radiomics software	Non-radiomics cofactors
Xie: 2021 [63]	ESCC 2007–2016	CT	Inst 1: 120KVp, 200-400 mA, 2.5 mm slices; Inst 2: 120KVp, 200-300 mA, 5 mm	nCRT	65 (train) 41 (test)	HF	PyRadiomics	Genetic
Beukinga: 2021 [28]	ESCC and EAD 2010–2018	PET/ CT	Gaussian filter of 6.5 mm in full-width at half-maximum	nCRT	96 (ESCC: 88 EAD: 8)	HF	In-house (Matlab V2018a)	Clinical factors, HER2 and CD44
Hu: 2021 [64]	ESCC 2007–2018	CT	Same as Hu:2020	nCRT	161 (train) 70 (test)	HF and DLF	PyRadiomics (V2.1.2)	No
Wang: 2021 [31]	ESCC and EAD 2012–2018	CT	120KVp, 200 mA, 3 mm	dCCRT	200 (train, ESCC: 189, EAD: 11) 200 (val., ESCC: 195, EAD: 5)	HF	3D Slicer (V4.8.1)	Clinicopathological, dosimetrics, and hematological
Li: 2020 [36]	ESCC Train 2009–2013 Val. 2015–2018	PET/CT	Voxel size: 4 × 4 × 5 mm3	dCCRT	152 (train) 32 (val.)	HF	PyRadiomics (V2.0.1)	Clinical and classical PET
Xie: 2020 [58]	ESCC 2008–2014	CT	120 kV, 180–280 mA, 3 mm	CCRT	57	HF	IBEX (V1.0β)	Clinical factors
Hu: 2020 [29]	ESCC 2007–2018	CT	120 kV, 200–400 mA 2.5 mm (inst 1) 5 mm (inst 2) voxel sizes: 1 × 1 × 5 mm3	nCRT	161 (train) 70 (test)	HF	PyRadiomics (V3.0)	No
Luo: 2020 [41]	ESCC 2013–2015	CT	120 kV, 120 mAs, 5 mm	dCCRT	160 (train) 66 (val.)	HF	3DSlicer (V4.10.2)	Clinical factors
Li: 2020 [54]	ESCC 2012–2019	CT	120 kV/140 kV, 140–300 mA, 5 mm	nCRT	121	HF	IBEX	Clinical factors
Zhang: 2020 [47]	EAD 2010–2016	PET/ CT	120 kVp, 20–200 mA	surgery alone, neoadjuvant chemotherapy, and nCRT	190	HF	Matlab	Clinical factors
Du: 2020 [42]	ESCC 2017–2019	CBCT	125 kVp, 80 mA, 13 ms, 680mAs, pixel size: 384 × 384, 2.5 mm, half-fan CBCT	dCCRT or definitive radiotherapy	67 (train) 29 (val.)	HF	3D Slicer (V4.10.2)	Clinical and dosimetrics
Foley: 2019 [35]	ESCC and EAD 2010–2015	PET/ CT	Same as Foley:2018	Same as Foley:2018	46 (external val.)	HF	In-house (Matlab)	Clinical and classical PET
Xie: 2019 [57]	ESCC Train 2012–2016 Val. 2008–2011	CT	Inst 1: 120 kVp, 406 mAs, 3–5 mm Inst 2: 120 kVp, 150 mAs, 3–8 mm Voxel size: 1 × 1 × 5 mm3	dCCRT	87 (train) 46 (val.)	HF	In-house (Matlab 2015b)	No
Wang: 2019 [60]	ESCC Train 2012–2016 Val. 2004–2014	CT	120 kV, 180-280 mA, 3 mm	CCRT and RT alone	83 (train) 98 + 283 (val.)	HF	IBEX (V1.0β)	Clinical
Chen: 2019 [30]	ESCC 2011–2017	PET/ CT	PET scanner: 120 kV, 12 mA, 3.75 mm	dCCRT	44	HF	CGITA	Clinical and classical PET
Yan: 2019 [32]	ESCC 2013–2017	CT	120kVp, 4 mm	nCRT	32	HF	CUBETAB (Matlab V2017b)	None
Yang: 2019 [33]	ESCC 2012–2016	CT	120 kVp, pixel size: 1.46 mm, 5 mm	nCRT	44 (train) 11 (test)	HF	3DSlicer (V4.8.1)	Clinical factors
Jin: 2019 [48]	ESCC, EAD, and Small cell 2012–2015	CT	120 kV, 180–280 mA, 3 mm	CCRT	94 (ESCC: 92, EAD: 1, Small cell: 1)	HF	IBEX	Clinical and dosimetrics
Foley: 2018 [34]	ESCC and EAD Train 2010–2014 Val. 2014–2015	PET/ CT	PET: 120 kVp, 20–200 mA	Multiple treatments incl nCRT and dCCRT	302 (train, ESCC: 65 EAD: 237) 101 (val., ESCC: 79 EAD: 22)	HF	In-house (Matlab)	Clinical and classical PET
Larue: 2018 [43]	ESCC (*n* = 46) and EAD (*n* = 193) 2010–2016	CT	Inst 1: 120 kV, 2.5–5 mm Inst 2: 120 kV, 1–3 mm Voxel size: 1 × 1 × 3 mm3	nCRT	165 (train) 74 (val.)	HF	In-house (Matlab)	Clinical
Beukinga: 2018 [49]	ESCC and EAD 2014–2017	PET/CT	80–120 kV, 20–35 mAs, 5 mm	nCRT	73 (ESCC: 8, EAD: 65)	HF	In-house (Matlab V2014b)	Clinical and classical PET
Riyahi: 2018 [52]	ESCC and EAD 2006–2009	PET/CT	Same as Tan:2013	Same as Tan:2013	Same as Tan:2013	HF	Elastix and ITK toolbox	Classical PET features
Paul: 2017 [37]	n.r	PET/CT	Voxel size: 4 × 4 × 2mm3	CCRT	65	HF	n.r	Clinical and classical PET
Desbordes: 2017 [50]	ESCC and EAD 2006–2013	PET/CT	Voxel size: 4 × 4 × 2 mm3	CCRT	65 (ESCC: 57 EAD: 8)	HF	n.r	Clinical and classical PET
Nakajo: 2017 [59]	n.r. 2011–2013	PET/ CT	120 kV, 35–100 mA, 3.75 mm	CCRT	52	HF	In-house (Python)	classical PET features
Beukinga: 2017 [45]	ESCC and EAD 2009–2016	PET/CT	PET: 0.98 × 0.98 mm, 2 mm; CT: 0.98 × 0.98 mm, 3 mm	nCRT	97 (ESCC: 9, EAD: 88)	HF	n.r	Clinical and classical PET
Wakatsuki: 2017 [62]	ESCC and EAD 2008–2015	CT	120 kV, 5 mm	nCRT	50 (ESCC: 46, EAD: 4)	HF	Unnamed	Clinical and histopathologic
Hou: 2017 [53]	ESCC 2015–2016	CT	120 kV, 200–250 mAs, 2.5–3 mm, pixel size: 0.97 × 0.97 mm	dCCRT	37 (train) 12 (test)	HF	In-house (Matlab 2015a)	No
Yip: 2016 [61]	ESCC and EAD	PET/CT	n.r	nCRT	45 (ESCC: 1, EAD: 44)	HF	CGITA	Classical PET features
Rossum: 2016 [38]	EAD 2006–2013	PET/CT	CT: 120 kV, 300 mA, 3.75 mm, voxel size: 5.47 × 5.47 × 3.27 mm	nCRT	217	HF	IBEX	Clinical and classical PET
Ypsilantis: 2015 [46]	ESCC and EAD n.r	PET/CT	3.27 mm, pixel size: 4.7 × 4.7 mm	nCRT	107 (ESCC: 20, EAD: 86, Undefined: 1)	HF/DLF	n.r	No
Yip: 2014 [51]	ESCC and EAD 2005–2008	CT	120 kV, 180–280 mA, 3–5 mm	dCCRT	36 (ESCC: 26 EAD: 9 Not specified:1)	HF	TexRAD	Clinical
Zhang: 2014 [40]	ESCC and EAD 2006–2009	PET/ CT	Same as Tan:2013	nCRT	20 (ESCC: 3, EAD: 17)	HF	n.r	Clinical and classical PET
Tan: 2013 [44]	ESCC and EAD 2006–2009	PET/ CT	120 kV, 200 mA, 0.98 × 0.98 × 4 mm3 (CT) 4 × 4 × 4 mm3 (PET)	nCRT	20 (ESCC: 3, EAD: 17)	HF	n.r	Classical PET features
Hatt: 2013 [55]	ESCC and EAD 2004–2008	PET/ CT	120 kV, 100mAs (CT) PET voxel size: 4 × 4 × 4 mm3	CCRT	50 (ESCC: 36, EAD: 14)	HF	n.r	Classical PET features
Tan: 2013 [56]	ESCC and EAD 2006–2009	PET/ CT	Same as Tan:2013	nCRT	20 (ESCC: 3 EAD: 17)	HF	ITK	Classical PET features
Tixier: 2011 [39]	ESCC and EAD 2003–2008	PET/ CT	n.r	CCRT	41 (ESCC: 31 EAD: 10)	HF	n.r	Classical PET features

Abbreviations used in the table: *n.r.* not reported; *val.* validation; *ESCC* esophageal squamous cell carcinoma; *EAD* esophageal adenocarcinoma; *nCRT* neoadjuvant chemoradiotherapy; *CCRT* concurrent chemoradiotherapy; *dCCRT* definitive concurrent chemoradiotherapy; *RT* radiotherapy; *CT* computed tomography; *CBCT* cone-beam computed tomography; *HF* handcrafted features; *DLF* deep learning-based features

The majority of imaging modalities mentioned in the retrieved studies were PET (20/37) [28, 30, 34–40, 44–47, 49, 50, 52, 55, 56, 59, 61], CT (16/37) [29, 31–33, 41, 43, 48, 51, 53, 54, 57, 58, 60, 62–64], and one cone beam CT (CBCT) [42]. Although the search criteria included MRI, we did not locate any eligible study in our search.

More than half of the included studies (19/37) addressed nCRT [28–30, 33, 35, 38, 40, 43–47, 49, 52, 54, 56, 61–64]. The majority of patients included in 13 studies were treated specifically with radical CCRT [31, 32, 36, 39, 41, 42, 48, 50, 51, 53, 55, 58, 59]. In three studies, most patients were treated with CCRT, but the rest received a variety of different treatments depending on their situation [34, 57, 60]. There was one study that did not specify the intent of CCRT [37].

The number of patients reported in the included studies ranged from 20 [40, 44, 52, 56] up to 464 [60]. Three studies utilized deep learning [46, 53, 64] and all other studies used only handcrafted features with Cox proportional hazards, logistic regression (LR), linear regression, support vector machine (SVM), and random forest (RF) models.

There were a wide range of software tools used to extract radiomics features. The in-house codes were predominantly generated in Matlab and Python. The most commonly used [31, 33, 41, 42] free and open-source software package was 3D Slicer [66], which allowed for manual or semi-automatic ROI delineation followed by radiomics features extraction using its radiomics [67] plug-in. Studies using Python and 3D Slicer were almost exclusively based on the *PyRadiomics* library [67] developed by Griethuysen et al.

Five studies investigated exclusively radiomics features [29, 32, 46, 53, 57], while the other studies examined a combination of radiomics with non-radiomics features (most commonly, clinical factors). In this review, classical PET features were defined as intensity-related metrics such as standardized uptake value (SUV), metabolic tumor volume (MTV), and total lesion glycolysis (TLG). There were 8, 7, and 10 studies that combined radiomics with clinical features [33, 41, 43, 47, 51, 54, 58, 60], classical PET features [39, 44, 52, 55, 56, 59, 61], and both clinical and classical PET features [30, 34–38, 40, 45, 49, 50], respectively. Among more recently published studies, three included genes as features [28, 63, 64], two included clinical factors with dosimetric features [42, 48], one included histopathologic features [62], and one used a combination of clinicopathological, dosimetric, and hematological features [31].

### Overall characteristics of included studies

The model results from the included studies are summarized in Table 2 and additional details are added in Supplementary Material Table S2. Patient data were mostly retrospectively extracted (31/37). Only four studies re-analyzed prospectively collected data, which all originated in the CROSS clinical trial [35, 45, 47, 49]. Three studies used both prospective and retrospective data, where the prospective data were also re-analyzed from other clinical trials [35, 47, 63]. One study did not describe if the data used was retrospectively or prospectively derived [46].

**Table 2:** Summary of radiomics-based prediction model characteristics described in included studies

Ref	Data type	# of institution(s)	Predicted outcome(s)	# of events/# of samples	# of features (considered / in final model)	Type of model	Reported performance	Model calibration tested
Xie: 2021 [63]	R + P	2	DFS	Train: 21/28 Int. validation: 24/37 External test: 13/41	2553/8	Cox	(train, validation and external test) AUC = 0.912, 0.852, and 0.769 C-index = 0.869, 0.812, and 0.719	Yes
Beukinga: 2021 [28]	R	1	pCR after nCRT	Group 1: 21/96 Group 2: 9/43	101/2	LR	AUC = 0.685 and 0.857 (Best of group 1 and group 2)	Yes
Hu: 2021 [64]	R	2	pCR after nCRT	Train: 74/161 Test: 31/70	Handcrafted features: 851/7 Handcrafted combined with deep learning-based: n.r./14	SVM	Handcrafted model: AUC = 0.822, and 0.725 (train and test) Deep learning-based: AUC = 0.807–0.901, and 0.635–0.805 (train and test)	Yes
Wang: 2021 [31]	R	2	RP	Train: 45/200 Val.: 41/200	850/24	Linear regression	C-index = 0.975, and 0.921 (internal and external val.)	Yes
Li: 2020 [36]	R	2	OS, DFS, LC	n.r./184	DFS: 105/3 OS: 105/4 LC: 105/4	Cox	Clustering of OS: *p* < 0.0001	No
Xie: 2020 [58]	R	1	OS	1-year survival: 43/57	16/4	Cox	1-year and 2-year survival: AUC = 0.79	No
Hu: 2020 [29]	R	2	pCR after nCRT	Train: 74/161 Test: 31/70	Intratumoral: 1208/16 Peritumoral: 1036/8 Combined model: 7 (intra) and 6 (peri)	8 different types of models	Combined model AUC = 0.906, and 0.852 (train and test)	Yes
Luo: 2020 [41]	R	1	CR after CCRT	Train: 56/160 Val.: 22/66	851/7	LASSO-LR	AUC = 0.844, and 0.807 (train and val.)	No
Li: 2020 [54]	R	1	pCR after nCRT	51/121	405/18	LR	AUC = 0.84 (val.)	Yes
Zhang: 2020 [47]	R + P	2	Clinical lymph node staging	Train: 75/130 Val.: 35/60	154/9	LR	AUC = 0.82, and 0.69 (train and val.)	Yes
Du: 2020 [42]	R	1	RP	39/96	851/2	LR	AUC = 0.836, and 0.905 (train and val.)	Yes
Foley: 2019 [35]	R + P	2	OS	External val.: 26/46	16/3	Cox	X2 = 1.27, df = 3, *p* = 0.74 (Kaplan–Meier)	Yes
Xie: 2019 [57]	R	2	OS	Train: 26/87 Val.: 9/46	548/7	Cox	AUC = 0.811 (Train) AUC = 0.805 (Val.)	No
Wang: 2019 [60]	R	3	OS PFS	Train: 23/83, Val.1: 18/98, Val.2: 53/283 Train: 21/83, Val.1: 8/98, Val.2 36/283	1/1	Cox	(Train, Val. 1 and 2) OS: C-index = 0.64, 0.61, and 0.58 PFS: C-index = 0.66, 0.60, and 0.57	No
Chen: 2019 [30]	R	1	pCR after nCRT, DFS, OS	nCRT response: 17/42	nCRT response 23/1	n.r	Clustering response to nCRT: *p* = 0.009	No
Yan: 2019 [32]	R	1	CR after RT survival	CR: 22/32	CR: 10/4 Survival: 10/2	n.r	RT response: *p* < 0.0001 Survival: *r* = 0.9917, *p* = 0.0001	No
Yang: 2019 [33]	R	1	pCR after nCRT	Train: 19/44 Test: 4/11	1030/5 (Model 1), 6 (Model 2/3)	LR	Model 1(bin size = 32): 0.86, and 0.79 (train and test)	No
Jin: 2019 [48]	R	1	response to CCRT	58/94	42/n.r	SVM, XGBoost	AUC = 0.689	No
Foley: 2018 [34]	R	1	OS	Train: 70/302 Test: 43/101	16/3	Cox	X2 143.14, df 3, *p* < 0.001(Train) X2 20.621, df 3, *p* < 0.001(Val.)	No
Larue: 2018 [43]	R	2	OS	Train: 67/165 Val.: 25/74	1049/40	RF	AUC = 0.69 (Train) AUC = 0.61 (Val.)	No
Beukinga: 2018 [49]	P	1	pCR after nCRT	16/73	113/6	LASSO-LR	AUC = 0.82 and 0.81 (train and val.)	Yes
Riyahi: 2018 [52]	R	1	pCR/mRD after nCRT	9/20	664/2	SVM-LASSO	AUC = 0.94 ± 0.05	No
Paul: 2017 [37]	R	1	CR after CCRT, OS	CR: 41/65 OS: 16/65	CR: 45/9 OS: 45/8	RF	CR: AUC = 0.823 ± 0.032 OS: AUC = 0.750 ± 0.108	No
Desbordes: 2017 [50]	R	1	CR after CCRT, 3-year OS	CR: 41/65 OS: 24/65	45/1	RF	CR: AUC = 0.836 ± 0.105 OS: AUC = 0.822 ± 0.059	No
Nakajo: 2017 [59]	R	1	CR/RP after CCRT, PFS, OS	CR: 18/52	CR 6/2 PFS and OS 6/0	Cox	CR: AUC = 0.75 PFS and OS: *p* < 0.001	No
Beukinga: 2017 [45]	P	1	pCR after nCRT	19/97	140/20	LR	AUC = 0.78, and 0.74 (train and val.)	Yes
Wakatsuki: 2017 [62]	R	1	response to nCRT	17/50	1/1 CT number	LR	AUC = 0.73, *p* = 0.009	No
Hou: 2017 [53]	R	1	CR/PR after CCRT	Train: 26/37 Test: 7/12	SVM: 214/9 ANN: 214/7	SVM, ANN	ANN: accuracy = 0.972, and 0.917; AUC = 0.927, and 0.800 (train and test) SVM: accuracy = 0.891, and 0.667; AUC = 0.818, and 0.600 (train and test)	No
Yip: 2016 [61]	R	1	response to nCRT	30/45	3/3	n.r	AUC = 0.72‒0.78	No
Rossum: 2016 [38]	R	1	pCR after nCRT	59/217	78/9	LR	C-index = 0.82 (apparent) C-index = 0.77 (corrected)	Yes
Ypsilantis: 2015 [46]	n.r	1	response to nCRT	38/107	85/n.r	LR, gradient boosting, RF, SVM, CNN	Accuracy: 73.4 ± 5.3	No
Yip: 2014 [51]	R	1	OS	5/36	6/4	Cox	AUC = 0.802	No
Zhang: 2014 [40]	R	1	pCR/mRD after nCRT	9/20	137/14	SVM, LR	AUC = 1 (no misclassifications)	No
Tan: 2013 [44]	R	1	pCR/mRD after nCRT	9/20	16 + 19/2 + 16	n.r	Texture feature: AUC = 0.83, p = 0.01; histogram distances: AUC = 0.78–0.89, *p* = 0.04	No
Hatt: 2013 [55]	R	1	CR/PR after CCRT	36/50	9/9	n.r	(best) AUC = 0.90	No
Tan: 2013 [56]	R	1	pCR/mRD after nCRT	10/20	33/2	n.r	(best) AUC = 0.85	No
Tixier: 2011 [39]	R	1	CR/PR after CCRT	CR: 9/41 PR: 21/41	38/4	n.r	Sensitivity: 76–92% Specificity: 56–91%	No

Abbreviations used in the table: *#* number; *R* retrospective; *P* prospective; *OS* overall survival; *DFS* disease-free survival; *PFS* progression-free survival; *LC* local control; *pCR* complete pathologic response; *mRD* microscopic residual disease; *SVM* support vector machine; *RF* random forest; *RT* radiotherapy; *CR* complete responders; *PR* partial responders; *LASSO* least absolute shrinkage and selection operator; *LR* logistic regression; *XGBoost* extreme gradient boosting; *ANN* artificial neural network; *CNN* convolutional neural network; *AUC* area under the receiver operating characteristic curve; *RT* radiotherapy; *nCRT* neoadjuvant chemoradiotherapy; *CCRT* concurrent chemoradiotherapy; *RP* radiation pneumonitis

There were few multi-institute studies in general. The majority of studies (27/37) were performed within a single institution. Nine studies incorporated data from two distinct institutes, and one study incorporated data from three distinct institutes.

Study endpoints were broadly classified into three categories: (1) prognosis (9/37), such as overall survival (OS), progression-free survival (PFS), and disease-free survival (DFS); (2) treatment response (20/37), such as prediction of complete/partial response after radical CCRT and pathology complete response (pCR) after nCRT; and (3) others, such as prediction of lymph node status [47] and radiation pneumonitis (RP) [31, 42]. There were five studies that reported both prognosis and treatment response prediction [30, 32, 37, 50, 59].

The number of events of the included studies ranged from 9 [52] to 113 [34], and the number of radiomics features in the final model ranged from only one [60, 62] up to 40 [43]. Overall, the number of events was small relative to the number of selected features. The number of positive events from studies predicting treatment-related side effects was overall much smaller than those predicting prognosis, which was consistent with real-world incidences.

The most frequently used model was Cox regression, followed by logistic regression. The most widely used machine learning approach was SVM (*n* = 7), but there was high heterogeneity in mathematical procedures. The deep learning architectures used were artificial neural networks (ANN) in one study [53] and convolutional neural networks (CNN) in two studies [46, 64], respectively.

Model performance had been summarized according to different study endpoints. For prognosis, some studies grouped patients by clustering only. Studies that reported the discriminative performance of the models had c-indices ranging from 0.64 [60] to 0.875 [63], and AUCs ranging from 0.69 [43] to 0.918 [63] in the training set. As expected, the discriminative performance overall decreased in the validation/test cohort, with c-indices ranging from 0.57 [60] to 0.719 [63] and AUCs between 0.61 [43, 60] and 0.805 [57] in the validation/test set.

For treatment response, reported AUCs were from 0.685 [28] to 1.0 [40] in training set but decreased overall in the validation/test sets (AUCs 0.6 [53] to 0.852 [29]). AUCs in the training and validation sets for the prediction of lymph node metastases study were 0.82 and 0.69 [47], respectively, and the AUCs in the validation set for the prediction of RP study were 0.921 [31] and 0.905 [42]. Except for RP, the validation set AUCs were roughly in the range of 0.6–0.8. Only six studies performed model calibration, four of which used the Hosmer–Lemeshow test for goodness of fit [28, 45, 47, 49].

### Methodological quality of the included studies

Given the rapid advances in AI tools and radiomics expertise, we restricted the assessment of methodological quality of recent radiomics studies published in the last 5 years [28–38, 41–43, 45, 47–50, 52–54, 57–64]. Table 3 provides an overview of the distribution of methodological quality and reporting completeness of 30 recent studies. A detailed report of quality assessment by the authors has been provided in Supplementary Material Table S3.

**Table 3:**
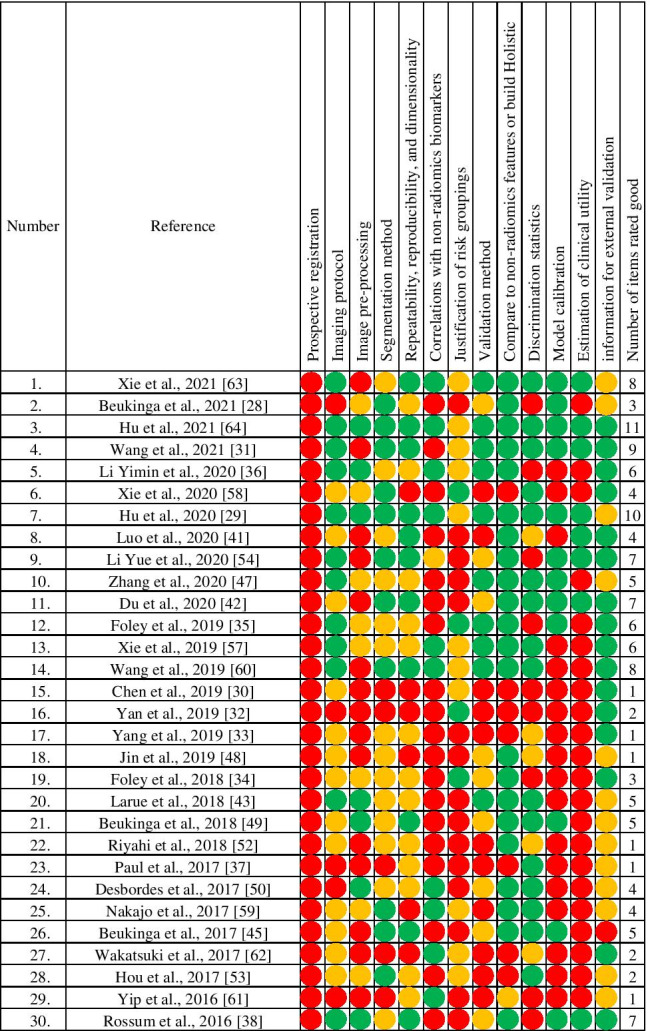
Assessment of methodological quality of included studies

Red circle: Poor rating, Yellow circle: Moderate rating, Green circle: Good rating

No study had been prospectively registered prior to commencement of the radiomics analysis. Among the 13 methodological items considered, around one-third of the studies reported essential details about image acquisition settings (12/30 rated good), digital image preprocessing (only 7/30 rated good), and how ROIs were derived (11/30 rated good).

In terms of feature selection, 11/30 studies evaluated repeatability/reproducibility of individual features and/or performed well-justified dimensionality reduction prior to fitting the final model. Ten studies tested the relationship between radiomics and non-radiomic features; out of which, 4 showed an association between radiomic features and PET uptake measures [36, 50, 59, 61], another 4 showed the relationship between radiomics and gene expression [29, 62–64], and the next 2 evaluated correlation between radiomics and clinical features [57, 60].

For elements related to reporting model performance, discrimination metrics in training and validation, with confidence intervals, were mostly reported well (16/30 studies), but fewer studies also included a check for model calibration (12/30 studies). Half (15/30 studies) defined clinically appropriate risk groupings and four studies used median [32, 58] or quartiles [34, 35] as risk group cut-offs, but two studies did not specify how risk groups were obtained [36, 60]. A few (5/30 studies) used ROC curves to obtain optimally-tuned cut-offs (e.g., Youden index).

For model validation, we found 10/30 studies used multi-institutional data, and 9/30 used internal cross-validation with some form of random splits of data, of which 5/30 studies used bootstrap methods ranging from 1000 to 20,000 replicates.

In regard to clinical impact, relatively few studies (8/30) estimated the clinical impact of their models, including use of decision curve analysis. Only 3 studies reported on all of model discrimination, model performance, and clinical utility in the same time [31, 42, 63]. The majority of radiomics studies (22/30) had been compared against non-radiomics models and/or constructed combined models.

As for documentation of the final prognostic model to a degree that permitted independent external validation, only 16/30 studies were rated as good. One study failed to report on the features selected in the final model. However, none of these 30 studies made their models or analysis code available for download from an electronic repository.

We further observed that methodological aspects among recent studies for predicting prognosis were generally somewhat better than for studies aiming to predict treatment response. Eleven studies were rated “good” for at least 6 out of 13 assessment items, whereas five studies of PFS or/and OS [35, 36, 57, 60, 63], four studies predicted treatment response (pCR after nCRT) [29, 38, 54, 64], and two studies predicting RP [31, 42] were of similar ratings. The best rating among these studies was scored “good” for 11 out of 13 items [64].

**Fig. 2:**
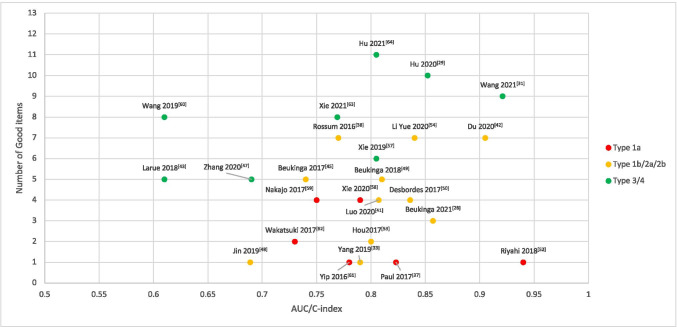
Reported AUC/C-index of the included studies with number of good items were classified by Transparent Reporting of a multivariable prediction model for Individual Prognosis Or Diagnosis (TRIPOD). Type 1a: development only; type 1b: development and validation using resampling; type 2a: random split-sample development and validation; type 2b: non-random split-sample development and validation; type 3: development and validation using separate data; type 4: validation only

Figure 2 visually summarizes the headline reported discrimination metric (AUC or c-indices) with the number of methodological items rated “good” in this review. Additionally, we have color-coded the dots to correspond to the TRIPOD type of study. A small number of methodologically strong studies near the top of the figure suggest a discriminative performance around 0.8 to 0.92 for radiomics prognostic models in EC, followed by a wider scatter of performance metrics for models of lower methodological rigor ranging from 0.61 up to 0.94. Interestingly, this overview found no models with a discriminative index lower than 0.6. The highest reported discrimination metric however coincides with a study of questionable methodological robustness. Overlaid above this, there is a clear trend of TRIPOD type 3 or 4 study designs obtaining higher methodological robustness ratings than TRIPOD types 1B, 2A, or 2B, with TRIPOD type 1A study designs tending towards the lower methodological ratings. A detailed description of different types of prediction model studies covered by TRIPOD statement can be found in the Reference [68].

## Discussion

This systematic review summarized the basic characteristics and the reported results of radiomics studies predicting clinical outcomes after CCRT in EC, and assessed the methodological quality of recent studies. The included studies focused on the prediction of treatment response and side effects to neoadjuvant and definitive CCRT, and prognosis. Prediction models were constructed by using either handcrafted or deep learning-based radiomics features. Although a few methodologically robust studies have reported promising results and have demonstrated the potential to be adopted as clinical practice tools, the methodological quality of a sizable number of studies remains suboptimal. Future studies have significant room for improvement in terms of more complete reporting of essential details of the modelling work, more robust methods in construction of the model, and better documentation of the final model such that independent external validation can be easily performed.

The results of this review showed that more and more researchers are investigating radiomics for prediction of nCRT response in EC. Most of these studies used pCR as an endpoint, with AUC ranging from 0.74 [45] to 0.857 [28]. However, one of the most significant shortcomings is lack of independent validation. We think that more attention should be given to testing the wider generalizability of the models through independent external validation. In addition, the difference in radiotherapy and chemotherapy regimens used in studies will also affect the probability of achieving pCR. Although some studies have combined clinical parameters with radiomics, the effect of different treatment regimens on the predictive power of the final model has not yet been investigated in detail.

Li et al. [54] demonstrated that radiomics combined with clinical factors has a superior discriminative performance and a better goodness-of-fit than the clinical model. According to Van et al. [38], the addition of comprehensive PET features improves the predictive power of the model compared to using only clinical features. Based on the results of the studies included in this review, it can be concluded that the predictive power of a multidimensional predictive model is usually higher than that of a predictive model built using a single type of data.

Hu et al. [29] showed that peritumoral CT handcrafted features were less robust than the intratumoral features, and the predictive power of the model could be improved by combining peritumoral and intratumoral features. This study also included a radiogenomics analysis to explain the association of peritumoral tissue with pCR from the perspective of immune microenvironment. This result gives us an indication that the definition of ROI should be further explored. Furthermore, Hu et al. [64] conducted a deep learning study that used the same cohort of data to extract features by using six CNN models with AUCs in the range of 0.635–0.805, which demonstrated that deep learning-based radiomics also have the ability to predict the response to nCRT.

Three other studies defined endpoints as greater than 30% reduction of tumor [48], Mandard grades 1–3 [62], and downstaging [61] and obtained moderate predictive efficacy (AUC range was 0.689–0.78). We can see that a radiomics-based model can screen out not only the patients who are very sensitive to nCRT, which refers to those who can achieve pCR, but also the patients who have partial remission.

In countries such as China and Japan, clinical guidelines recommend concurrent chemoradiotherapy as the standard of care, but fewer patients in these countries receive this type of treatment in clinical practice compared to Western countries. The reason for this may be related to the different tolerances and responses to side effects in different ethnic groups [69]. However, it might also be related to genetics, since a number of studies [70–72] revealed a correlation between gene single nucleotide polymorphism and the intrinsic radiosensitivity of the lung to radiation. Therefore, if rare side effects associated with concurrent chemoradiotherapy of the esophagus can be accurately predicted, it may be additionally helpful to improve the treatment outcome and the quality of patient survival, as well as to assist in clinical decision-making.

Accurately predicting patient prognosis is still a challenging task, and some studies have used radiomics for predicting endpoints such as OS, PFS, and DFS, but the results vary widely, with C-index/AUC ranging from 0.57 [60] to 0.822 [50]. These studies used retrospective data, and one of the most fundamental problems is that the accuracy of follow-up with prognosis as an endpoint cannot always be obtained. In general, the current studies for prognostic prediction are pilot investigations, and adding more dimensions such as clinical parameters and genetic information can improve the predictive power of model.

With our 13-point methodological assessment criteria, we must emphasize that we are not proposing that some models are intrinsically “better” or “worse.” The primary purpose of the critical appraisal was to understand which of these reported model results have a high likelihood of being successfully reproduced independently elsewhere, and thus have higher change of wide clinical generalizability. Both reproducibility and generalizability are essential aspects of our estimation of methodological robustness.

It would have been ideal if data collection and a statistical analysis protocol of radiomics modelling studies could have been prospectively registered, but there is presently no widely held consensus on where such protocols or modelling studies might be registered in advance. We recommend that biomedical modelling registries (e.g., AIMe registry [73]) should be given more attention by the radiomics community, so that there exists an opportunity for collaboration, review, and advice for improvement prior to commencing a radiomics study.

The reviewed studies paid attention to imaging settings, ROI definition, discrimination metrics, and comparison of radiomics with non-radiomics predictors; however, relatively few studies gave the same degree of attentiveness to (i) documenting image pre-processing steps if any were used, (ii) clearly defining and justifying the clinical relevance of risk groupings, (iii) testing model calibration, and (iv) estimating the clinical impact of the model, for example, by decision curve analysis. We recommend that additional attention be paid to the aforementioned aspects by future researchers and journal editors.

Independent validation remains one of the key areas in which future radiomics modelling studies in EC could be significantly improved; our review found that the vast majority (27/30 studies) comprised solely of single-institutional datasets. Reporting of selected features in the final model together with regression coefficients would aid reproducibility testing of such models. In cases where a regression model has not been used, we recommend that models should be made openly accessible to download, or an online calculator of risk scores should be provided, to allow other researchers to independently externally validate using new datasets.

Adoption of standards and guidelines are expected to have an overall positive effect on widespread generalizability and external validity. If an option for prospective image collection for radiomics study exists, we recommend fully standardized image acquisition and reconstruction guidelines such as the EANM Research Limited (EARL) [74], but we also acknowledge that (for the present time) the vast majority of images available for radiomics study consist of retrospectively extracted data from routine care procedures. In addition to standardizing radiomics feature definitions, the imaging biomarker standardization initiative (IBSI) [75] advises reporting of patient handling, image acquisition, image pre-processing, feature extraction, and model building; hence, we also recommend this when reporting on radiomics analyses.

Studies reviewed were consistent such that the event rate was low compared to the number of possible model parameters considered (before feature selection/dimensionality reduction). This was especially true for models with treatment side effects as the primary outcome. Increasing the sample size and synthetically enhancing data diversity are two intuitive approaches that may be considered in the future. A growing number of domain generalization techniques are emerging from the deep learning field, such as domain adaptation [76] and meta-learning [77] that could assist the latter approach. However, the more immediate solution remains the former, and an option may be to make multi-institutional data publicly accessible in a centralized repository such as The Cancer Imaging Archive (TCIA). Alternatively, privacy-preserving federated learning [78] (also known as distributed learning) may be a feasible solution for modelling private data between institutions without physically exchanging individual patient data. Federated learning has been shown to be feasible in the radiomics domain [79, 80], and also for EC in particular [81].

Based on a small number of methodologically robust studies, we estimated the state of the art prognostic performance for radiomics models in EC to be in the ballpark of 0.85. There was indeed a correlation between our methodological assessment items with TRIPOD type of study, which is in agreement with a systematic review in lung cancer [25]. While we noted no studies published with a discriminative index below 0.60, we cannot at the present moment conclude whether or not this is a sign of publication bias; to effectively do this, we would need a prospective registry of modelling studies, as mentioned previously. This has been the widely adopted standard for epidemiological clinical studies (such as randomized controlled trials) as a means of incentivizing research transparency and detecting the presence of publication bias. Hence, we re-iterate our recommendation that the community should come to a consensus about a prospective registry for biomedical modelling studies.

Only a small number of studies at the present time addressed deep learning-based radiomics; however, we would expect this number to grow rapidly in the future. Different studies suggest that discriminative performance of deep learning models is superior to models based only on handcrafted features; however, it remains difficult to interpret the significance of deep learning features when applied to a specific clinical case. Explainable and interpretable deep learning is presently an active area of technical development, and we have seen some use of “attention mapping” (e.g., Grad-CAM [82]) to indicate which region of the image appears to influence the discrimination strongly. Additionally, research is also required to determine the relationship between image-based features and biological processes that may underpin the observed clinical outcomes.

We may note a number of limitations of the current systematic review that could potentially be addressed in some future work. First, we were not able to perform a quantitative meta-analysis due to the high heterogeneity of the mathematical procedures, even among related types of clinical outcome. Instead, we attempted a visual synthesis of reported model performance versus methodological robustness and TRIPOD study design (see Fig. 2). Secondly, we may have been able to detect more studies by searching in grey literature for non-peer reviewed work; however, we did not expect studies of high methodological quality to appear from those sources. On the other hand, it may have been possible to detect works where the model’s discriminative performance was between 0.5 and 0.6, whereas anything below 0.6 appears to be absent in our eligible articles. Thirdly, while we made our best possible attempt at evaluating methodological procedure with objective criteria, independent raters, and then combined consensus, some residual amount of subjectivity and debatable result of assessment may still persist; we have provided additional detailed notes in the supplementary material regarding methodology and tried to make our evaluations as transparent as possible. Lastly, we introduced some inclusion bias by only allowing full-text articles in the English language. This was done for the purely pragmatic reason that all authors of this review understood English and that such selected material will be accessible/understandable to readers of the present review, should they wish to inspect the individual papers by themselves.

## Conclusions

We summarized the available studies applying radiomics in predicting clinical outcomes of esophageal cancer patients who received concurrent chemoradiotherapy. Furthermore, the methodological quality of the included studies was analyzed to further improve the predictive power of radiomics and unlock the process of translation to clinical applications. Due to the limitations of inappropriate methodologies, incomplete and unclear reporting of information in radiomics model development and validation phases, the clinical application of radiomics has been impeded. The current systematic review pointed out these issues and provided our recommendations to increase generalization, biological interpretation, and clinical utility of a radiomics model.

## Supplementary Information

Below is the link to the electronic supplementary material.

Supplementary file1 (DOCX 60 KB)

## Data Availability

Primary data cited in the review are openly available in MEDLINE (PubMed) and Embase (Ovid) databases.
