# Supplementary material for: Methodological quality of machine learning-based quantitative imaging analysis studies in esophageal cancer: a systematic review of clinical outcome prediction after concurrent chemoradiotherapy
Source: Eur J Nucl Med Mol Imaging. 2021 Dec 23;49(8):2462–81. doi: 10.1007/s00259-021-05658-9 (PMC9206619; doi:10.1007/s00259-021-05658-9)
Supplement: Supplementary file 1 — Supplementary file1 (DOCX 60 KB) [file 259_2021_5658_MOESM1_ESM.docx]

**Supplementary materials**

Table S1: Systematic search

Table S2: Detailed image acquisition and model characteristics

Table S3: Assessment of methodological quality of included studies

**Supplementary Table 1: Systematic search**

1. Medline

((("Validat"[All Fields] OR (("predict"[All Fields] OR "predictabilities"[All Fields] OR "predictability"[All Fields] OR "predictable"[All Fields] OR "predictably"[All Fields] OR "predicted"[All Fields] OR "predicting"[All Fields] OR "prediction"[All Fields] OR "predictions"[All Fields] OR "predictive"[All Fields] OR "predictively"[All Fields] OR "predictiveness"[All Fields] OR "predictives"[All Fields] OR "predictivities"[All Fields] OR "predictivity"[All Fields] OR "predicts"[All Fields]) AND "ti"[All Fields]) OR "Rule"[All Fields] OR (("predict"[All Fields] OR "predictabilities"[All Fields] OR "predictability"[All Fields] OR "predictable"[All Fields] OR "predictably"[All Fields] OR "predicted"[All Fields] OR "predicting"[All Fields] OR "prediction"[All Fields] OR "predictions"[All Fields] OR "predictive"[All Fields] OR "predictively"[All Fields] OR "predictiveness"[All Fields] OR "predictives"[All Fields] OR "predictivities"[All Fields] OR "predictivity"[All Fields] OR "predicts"[All Fields]) AND ("outcome"[All Fields] OR "outcomes"[All Fields] OR ("risk"[MeSH Terms] OR "risk"[All Fields]) OR ("model"[All Fields] OR "model s"[All Fields] OR "modeled"[All Fields] OR "modeler"[All Fields] OR "modeler s"[All Fields] OR "modelers"[All Fields] OR "modeling"[All Fields] OR "modelings"[All Fields] OR "modelization"[All Fields] OR "modelizations"[All Fields] OR "modelize"[All Fields] OR "modelized"[All Fields] OR "modelled"[All Fields] OR "modeller"[All Fields] OR "modellers"[All Fields] OR "modelling"[All Fields] OR "modellings"[All Fields] OR "models"[All Fields]))) OR (("history"[MeSH Terms] OR "history"[All Fields] OR "histories"[All Fields] OR "history"[MeSH Subheading] OR ("variabilities"[All Fields] OR "variability"[All Fields] OR "variable"[All Fields] OR "variable s"[All Fields] OR "variables"[All Fields] OR "variably"[All Fields]) OR ("criteria s"[All Fields] OR "criterias"[All Fields] OR "standards"[MeSH Subheading] OR "standards"[All Fields] OR "criteria"[All Fields]) OR "Scor"[All Fields] OR ("characteristic"[All Fields] OR "characteristics"[All Fields]) OR ("diagnosis"[MeSH Subheading] OR "diagnosis"[All Fields] OR "findings"[All Fields] OR "diagnosis"[MeSH Terms] OR "finds"[All Fields] OR "signs and symptoms"[MeSH Terms] OR ("signs"[All Fields] AND "symptoms"[All Fields]) OR "signs and symptoms"[All Fields] OR "finding"[All Fields]) OR ("factor"[All Fields] OR "factor s"[All Fields] OR "factors"[All Fields])) AND ("predict"[All Fields] OR "predictabilities"[All Fields] OR "predictability"[All Fields] OR "predictable"[All Fields] OR "predictably"[All Fields] OR "predicted"[All Fields] OR "predicting"[All Fields] OR "prediction"[All Fields] OR "predictions"[All Fields] OR "predictive"[All Fields] OR "predictively"[All Fields] OR "predictiveness"[All Fields] OR "predictives"[All Fields] OR "predictivities"[All Fields] OR "predictivity"[All Fields] OR "predicts"[All Fields] OR ("model"[All Fields] OR "model s"[All Fields] OR "modeled"[All Fields] OR "modeler"[All Fields] OR "modeler s"[All Fields] OR "modelers"[All Fields] OR "modeling"[All Fields] OR "modelings"[All Fields] OR "modelization"[All Fields] OR "modelizations"[All Fields] OR "modelize"[All Fields] OR "modelized"[All Fields] OR "modelled"[All Fields] OR "modeller"[All Fields] OR "modellers"[All Fields] OR "modelling"[All Fields] OR "modellings"[All Fields] OR "models"[All Fields]) OR ("decision"[All Fields] OR "decision s"[All Fields] OR "decisions"[All Fields] OR "decisive"[All Fields] OR "decisively"[All Fields]) OR "Identif"[All Fields] OR "Prognos"[All Fields])) OR (("decision"[All Fields] OR "decision s"[All Fields] OR "decisions"[All Fields] OR "decisive"[All Fields] OR "decisively"[All Fields]) AND ("model"[All Fields] OR "model s"[All Fields] OR "modeled"[All Fields] OR "modeler"[All Fields] OR "modeler s"[All Fields] OR "modelers"[All Fields] OR "modeling"[All Fields] OR "modelings"[All Fields] OR "modelization"[All Fields] OR "modelizations"[All Fields] OR "modelize"[All Fields] OR "modelized"[All Fields] OR "modelled"[All Fields] OR "modeller"[All Fields] OR "modellers"[All Fields] OR "modelling"[All Fields] OR "modellings"[All Fields] OR "models"[All Fields] OR ("ambulatory care facilities"[MeSH Terms] OR ("ambulatory"[All Fields] AND "care"[All Fields] AND "facilities"[All Fields]) OR "ambulatory care facilities"[All Fields] OR "clinic"[All Fields] OR "clinic s"[All Fields] OR "clinical"[All Fields] OR "clinically"[All Fields] OR "clinicals"[All Fields] OR "clinics"[All Fields]) OR ("logistic models"[MeSH Terms] OR ("logistic"[All Fields] AND "models"[All Fields]) OR "logistic models"[All Fields]))) OR (("prognostic"[All Fields] OR "prognostical"[All Fields] OR "prognostically"[All Fields] OR "prognosticate"[All Fields] OR "prognosticated"[All Fields] OR "prognosticates"[All Fields] OR "prognosticating"[All Fields] OR "prognostication"[All Fields] OR "prognostications"[All Fields] OR "prognosticator"[All Fields] OR "prognosticators"[All Fields] OR "prognostics"[All Fields]) AND ("history"[MeSH Terms] OR "history"[All Fields] OR "histories"[All Fields] OR "history"[MeSH Subheading] OR ("variabilities"[All Fields] OR "variability"[All Fields] OR "variable"[All Fields] OR "variable s"[All Fields] OR "variables"[All Fields] OR "variably"[All Fields]) OR ("criteria s"[All Fields] OR "criterias"[All Fields] OR "standards"[MeSH Subheading] OR "standards"[All Fields] OR "criteria"[All Fields]) OR "Scor"[All Fields] OR ("characteristic"[All Fields] OR "characteristics"[All Fields]) OR ("diagnosis"[MeSH Subheading] OR "diagnosis"[All Fields] OR "findings"[All Fields] OR "diagnosis"[MeSH Terms] OR "finds"[All Fields] OR "signs and symptoms"[MeSH Terms] OR ("signs"[All Fields] AND "symptoms"[All Fields]) OR "signs and symptoms"[All Fields] OR "finding"[All Fields]) OR ("factor"[All Fields] OR "factor s"[All Fields] OR "factors"[All Fields]) OR ("model"[All Fields] OR "model s"[All Fields] OR "modeled"[All Fields] OR "modeler"[All Fields] OR "modeler s"[All Fields] OR "modelers"[All Fields] OR "modeling"[All Fields] OR "modelings"[All Fields] OR "modelization"[All Fields] OR "modelizations"[All Fields] OR "modelize"[All Fields] OR "modelized"[All Fields] OR "modelled"[All Fields] OR "modeller"[All Fields] OR "modellers"[All Fields] OR "modelling"[All Fields] OR "modellings"[All Fields] OR "models"[All Fields]))) OR ("stratification"[All Fields] OR "stratifications"[All Fields] OR ("roc curve"[MeSH Terms] OR ("roc"[All Fields] AND "curve"[All Fields]) OR "roc curve"[All Fields]) OR ("discriminabilities"[All Fields] OR "discriminability"[All Fields] OR "discriminable"[All Fields] OR "discriminably"[All Fields] OR "discriminance"[All Fields] OR "discriminant"[All Fields] OR "discriminants"[All Fields] OR "discriminate"[All Fields] OR "discriminated"[All Fields] OR "discriminates"[All Fields] OR "discriminating"[All Fields] OR "discrimination, psychological"[MeSH Terms] OR ("discrimination"[All Fields] AND "psychological"[All Fields]) OR "psychological discrimination"[All Fields] OR "discrimination"[All Fields] OR "discriminations"[All Fields] OR "discriminative"[All Fields] OR "discriminatively"[All Fields] OR "discriminator"[All Fields] OR "discriminators"[All Fields]) OR ("discriminabilities"[All Fields] OR "discriminability"[All Fields] OR "discriminable"[All Fields] OR "discriminably"[All Fields] OR "discriminance"[All Fields] OR "discriminant"[All Fields] OR "discriminants"[All Fields] OR "discriminate"[All Fields] OR "discriminated"[All Fields] OR "discriminates"[All Fields] OR "discriminating"[All Fields] OR "discrimination, psychological"[MeSH Terms] OR ("discrimination"[All Fields] AND "psychological"[All Fields]) OR "psychological discrimination"[All Fields] OR "discrimination"[All Fields] OR "discriminations"[All Fields] OR "discriminative"[All Fields] OR "discriminatively"[All Fields] OR "discriminator"[All Fields] OR "discriminators"[All Fields]) OR ("c basel"[Journal] AND ("statistic"[All Fields] OR "statistic s"[All Fields] OR "statistical"[All Fields] OR "statistically"[All Fields] OR "statistics"[All Fields])) OR ("c basel"[Journal] AND ("statistic"[All Fields] OR "statistic s"[All Fields] OR "statistical"[All Fields] OR "statistically"[All Fields] OR "statistics"[All Fields])) OR ("area under curve"[MeSH Terms] OR ("area"[All Fields] AND "under"[All Fields] AND "curve"[All Fields]) OR "area under curve"[All Fields] OR ("area"[All Fields] AND "under"[All Fields] AND "curve"[All Fields]) OR "area under the curve"[All Fields]) OR "auc"[All Fields] OR ("calibrant"[All Fields] OR "calibrants"[All Fields] OR "calibrate"[All Fields] OR "calibrated"[All Fields] OR "calibrates"[All Fields] OR "calibrating"[All Fields] OR "calibration"[MeSH Terms] OR "calibration"[All Fields] OR "calibrations"[All Fields] OR "calibrator"[All Fields] OR "calibrators"[All Fields]) OR ("indicate"[All Fields] OR "indicated"[All Fields] OR "indicates"[All Fields] OR "indicating"[All Fields] OR "indicative"[All Fields] OR "indicatives"[All Fields] OR "indicators and reagents"[Pharmacological Action] OR "indicators and reagents"[MeSH Terms] OR ("indicators"[All Fields] AND "reagents"[All Fields]) OR "indicators and reagents"[All Fields] OR "indicator"[All Fields] OR "indicators"[All Fields] OR "indice"[All Fields] OR "indices"[All Fields]) OR ("algorithm s"[All Fields] OR "algorithmic"[All Fields] OR "algorithmically"[All Fields] OR "algorithmics"[All Fields] OR "algorithmization"[All Fields] OR "algorithms"[MeSH Terms] OR "algorithms"[All Fields] OR "algorithm"[All Fields]) OR ("multivariable"[All Fields] OR "multivariables"[All Fields] OR "multivariably"[All Fields] OR "multivariance"[All Fields] OR "multivariant"[All Fields] OR "multivariate"[All Fields] OR "multivariated"[All Fields] OR "multivariately"[All Fields] OR "multivariates"[All Fields] OR "multivariative"[All Fields])) OR ("predict*"[Title/Abstract] OR "predictive value of tests"[MeSH Terms] OR "scor*"[Title/Abstract] OR "observ*"[Title/Abstract] OR "observer variation"[MeSH Terms])) AND ((esophageal cancer[MeSH Terms]) OR (esophageal cancers[MeSH Terms]))) AND ((((((computed tomography[Text Word]) OR (CT[Text Word])) OR (magnetic resonance imaging[Text Word])) OR (MR*[Text Word])) OR (positron emission tomography[Text Word])) OR (PET[Text Word]))) AND ((((((((((radiomic[Text Word]) OR (radiomics[Text Word])) OR (textur*[Text Word])) OR (quantitative[Text Word])) OR (artificial intelligence[Text Word])) OR (AI[Text Word])) OR (deep learning[Text Word])) OR (shape[Text Word])) OR (feature[Text Word])) OR (features[Text Word])) Filters: Humans, English

1. Embase
2. ('esophageal cancer'/exp AND 'computed tomography'/exp OR 'ct' OR 'magnetic resonance imaging'/exp OR 'mr*' OR 'pos-itron emission tomography' OR 'pet') AND 'radiomic' OR 'radiomics' OR 'textur*' OR 'quantitative' OR 'artificial in-telligence' OR 'ai' OR 'deep learning'/exp OR 'shape' OR 'feature' OR 'features':ta,ab,ti
3. 'validat' OR (('predict' OR 'predictabilities' OR 'predictability' OR 'predictable' OR 'predictably' OR 'predicted' OR 'pre-dicting' OR 'prediction' OR 'predictions' OR 'predictive' OR 'predictively' OR 'predictiveness' OR 'predictives' OR 'predictivi-ties' OR 'predictivity' OR 'predicts') AND 'ti') OR 'rule' OR (('predict':ta,ab,ti OR 'predictabilities':ta,ab,ti OR 'predictability':ta,ab,ti OR 'predictable':ta,ab,ti OR 'predictably':ta,ab,ti OR 'predicted':ta,ab,ti OR 'predicting':ta,ab,ti OR 'prediction':ta,ab,ti OR 'predictions':ta,ab,ti OR 'predictive':ta,ab,ti OR 'predictively':ta,ab,ti OR 'predic-tiveness':ta,ab,ti OR 'predictives':ta,ab,ti OR 'predictivities':ta,ab,ti OR 'predictivity':ta,ab,ti OR 'predicts':ta,ab,ti) AND ('outcome':ta,ab,ti OR 'outcomes':ta,ab,ti OR 'risk':ta,ab,ti OR 'model':ta,ab,ti OR 'model s':ta,ab,ti OR 'modeled':ta,ab,ti OR 'modeler':ta,ab,ti OR 'modeler s':ta,ab,ti OR 'modelers':ta,ab,ti OR 'modeling':ta,ab,ti OR 'modelings':ta,ab,ti OR 'modelization':ta,ab,ti OR 'modelizations':ta,ab,ti OR 'modelize':ta,ab,ti OR 'modelized':ta,ab,ti OR 'modelled':ta,ab,ti OR 'modeller':ta,ab,ti OR 'modellers':ta,ab,ti OR 'modelling':ta,ab,ti OR 'modellings':ta,ab,ti OR 'models':ta,ab,ti))
4. #1 AND #2 AND ([article]/lim OR [article in press]/lim OR [data papers]/lim) AND [english]/lim

**Supplementary Table 2: Detailed image acquisition and model characteristics**

| **Number** | **Reference** | **Image acquisition scanner and PET data acquisition time** | **Details of treatment modality** | **Detailed report performance** |  |
| --- | --- | --- | --- | --- | --- |
|  | Xie et al., 2021 | Institution 1: Aquilion TSX-101A (Toshiba) or Discovery 750 HD (GE)  Institution 2: Discovery VCT, GE Healthcare | All patients were treated with nCRT followed by surgery | AUC= 0.912 and 0.918; C-index=0.869 and 0.875 (nomogram 1 and 2 in the Training set)  AUC=0.852 and 0.810; C-index=0.812 and 0.757 (nomogram 1 and 2 in the Internal test set)  AUC= 0.769 and 0.724; C-index=0.719 and 0.668 (nomogram 1 and 2 in the External test set) |  |
|  | Beukinga et al., 2021 | Biograph mCT-64 PET/CT (Siemens)  Images were acquired sixty minutes after tracer injection | All patients were treated with nCRT followed by surgery | AUC = 0.685 (Best of group 1)  AUC = 0.857 (Best of group 2) |  |
|  | Hu et al., 2021 | Same as Hu et al., 2020 | Same as Hu et al., 2020 | Handcrafted model: AUC=0.822 (training)  AUC/C-index=0.725, accuracy=67.1% (test)  Deep learning-based: AUC=0.807-0.901 (training)  AUC=0.635-0.805(test) |  |
|  | Wang et al., 2021 | Philips Brilliance Big Bore CT scanner | All patients treated with definitive CCRT | C-index=0.975 (0.953‒0.996, 95% CI, internal validation)  C-index=0.921 (0.876‒0.966, 95% CI, external validation) |  |
|  | Li et al., 2020 | PET scanner: institution 1: Discovery STE (GE) Data acquisition started 67 ± 22 min (range 50–140 min) after injection of 142–548 MBq FDG  institution 2: Gemini TF 16 Astonish (Philips)  Data acquisition started 71 ± 9 min (range 60–86 min) after injection of 236–248 MBq FDG | (Training) Patients received definitive CCRT  (Validation) 22 patients received definitive CCRT while 10 patients received preoperative CCRT | Clustering of OS: p<0.0001 |  |
|  | Xie et al., 2020 | Contrast CT: 16–detector row CT scanner (PHILIPS) | All patients were treated with CCRT | 1-year and 2-year survival: AUC=0.79 |  |
|  | Hu et al., 2020 | Contrast-enhanced CT  scanner: Aquilion TSX-101A (Toshiba)  Discovery VCT, GE Healthcare | All patients underwent nCRT followed by surgery | Intratumoral model AUC=0.881(training)  AUC=0.730 (95%CI,0.609-0.850, test)  Peritumoral model  AUC=0.895 (training)  AUC=0.734 (95%CI,0.614-0.854, test)  Combined model  AUC=0.906 (training)  AUC=0.852 (95%CI,0.753-0.951, test) |  |
|  | Luo et al., 2020 | GE Lightspeed 64-slice spiral CT | All patients were treated with definitive CCRT | AUC=0.844 (95% CI 0.779–0.897, training)  AUC=0.807 (95% CI 0.691–0.894, validation) |  |
|  | Li et al., 2020 | A variety of CT scanners | All patients were treated with nCRT followed by surgery | AUC = 0.84 (validation) |  |
|  | Zhang et al., 2020 | PET scanner: GE 690 scanner  Uptake time was 90min. | (Training) 130 patients receiving either surgery alone, neoadjuvant chemotherapy, or nCRT followed by surgery  (Validation) 60 patients who underwent nCRT | AUC=0.82 (95% CI 0.74–0.89, training)  AUC=0.69 (95% CI 0.54–0.82, validation) |  |
|  | Du et al., 2020 | On-board imager (OBI) system mounted on the Varian Trilogy medical linear accelerator | Patients received definitive CCRT or definitive radiotherapy | AUC=0.836 (0.700–0.918, 95% CI, training)  AUC=0.905 (0.799–1.000, 95% CI, validation) |  |
|  | Foley et al., 2019 | Same as Foley et al., 2018 | Training and internal validation same as Foley et al., 2018  (External validation) All patients treated with nCRT | X2=1.27, df=3, p=0.74 (Kaplan-Meier) |  |
|  | Xie et al., 2019 | Institution 1: Brilliance Big Bore CT scanner (Philips)  Institution 2: LightSpeed Pro 16 CT (GE) | The majority of patients received deﬁnitive CCRT, for patients with advanced ages or poor performance status, radiotherapy alone was delivered to these patients. | Results of 1-year and 2-year survival are omitted in this table  3-year survival: AUC=0.811 (95%CI, 0.670–0.952, training)  AUC=0.805 (95%CI ,0.638–0.973, validation) |  |
|  | Wang et al., 2019 | Institution 1(Training set): contrast CT 16 detector row CT scanner (PHILIPS) | Most patients (237) were treated with CCRT, others (227) were treated with radiotherapy alone | OS: C-index= 0.64 (0.55–0.73,95% CI, training) C-index= 0.6073 (0.53-0.68,95% CI, validation 1) C-index= 0.58 (0.54-0.62,95% CI, validation 2)  PFS: C-index=0.66(0.58–0.74,95% CI, training) C-index=0.60(0.54-0.67,95% CI, validation 1) C-index= 0.57(0.53-0.61,95% CI, validation 2) |  |
|  | Chen et al., 2019 | PET scanner: GE Discovery ST PET/CT unit  PET images were obtained between 40 and 60 min after injection of 18F-FDG (400 MBq) | All patients underwent nCRT followed by surgery | Clustering response to nCRT: p=0.009 |  |
|  | Yang et al., 2019 | Non-enhanced CT scanner: Philips Brilliance CT Big Bore Oncology Conﬁguration, Cleveland, OH | All patients underwent nCRT followed by surgery | Model 1(bin size=32): 0.86 (95% CI, 0.74-0.98, training) 0.79 (95% CI, 0.48-1.00, test)  Model 2(bin size=64): 0.84 (95% CI, 0.72-0.95, training) 0.75 (95% CI, 0.42-1.00, test)  Model 3(bin size=128): 0.84 (95% CI, 0.72-0.96, training) 0.71 (95% CI, 0.38-1.00, test) |  |
|  | Yan et al., 2019 | CT scanner: CT-on-rails (CTVision; Siemens) during daily CT-guided IGRT. | All patients were received radiotherapy, of which 2 patients were preoperative Radiotherapy, 42 patients were CCRT | RT response: coarseness P <0.0001, STD P=0.0007, entropy P=0.0003, strength P <0.0001  Survival: coarseness r=0.9572, P=0.0027, strength r = 0.9917, P=0.0001 |  |
|  | Jin et al., 2019 | Contrast CT: 16-detector row (Brilliance, Phillips) | All patients were received CCRT | AUC=0.689 |  |
|  | Foley et al., 2018 | PET scanner: GE 690 scanner  Uptake time was 90min | Patients underwent surgery alone, neoadjuvant chemotherapy or nCRT prior to surgery, definitive CCRT, or palliative therapy. | X2 143.14, df 3, p < 0.001(Training)  X2 20.621, df 3, p < 0.001(validation) |  |
|  | Larue et al., 2018 | Institution 1: General Electric LightSpeed RT16 (General Electric), Philips PQ5000 or Philips Gemini TF (Philips)  Institution 2: Siemens SOMATOM Sensation Open CT or Siemens Biograph 40 PET/CT scanner | All patients treated with nCRT followed by surgery | AUC= 0.69 (95% CI 0.61–0.77, Training)  AUC=0.61 (95% CI 0.47–0.75, Validation) |  |
|  | Beukinga et al., 2018 | Biograph mCT-64 PET/CT (Siemens)  Images were acquired sixty minutes after tracer injection | All patients were treated with nCRT followed by surgery | AUC=0.82(Training) AUC=0.81(validation) |  |
|  | Riyahi et al., 2018 | Same as Tan et al., 2013 | Same as Tan et al., 2013 | Sensitivity=94.4±0.08%, Specificity=91.8±0.06%, Accuracy=94.0±0.05%,  AUC=0.94±0.05 |  |
|  | Paul et al., 2017 | PET/CT Biograph Sensation 16 (Siemens). | All patients were treated with CCRT | Response to treatment: AUC=0.823±0.032  OS: AUC=0.750±0.108 |  |
|  | Desbordes et al., 2017 | PET scanner: Biograph1 Sensation 16 Hi-Rez device (Siemens)  Images were acquired 60 (±10) minutes after tracer injection | All patients were treated by CCRT, 14 patients followed by surgery | Response: AUC=0.836±0.105  OS: AUC=0.822±0.059 |  |
|  | Nakajo et al., 2017 | Discovery 600 M PET/CT system (GE)  Images were acquired sixty minutes after tracer injection | All patients were treated with CCRT | Response to treatment: AUC=0.75  PFS and OS: P <0.001 |  |
|  | Beukinga et al., 2017 | Biograph mCT 4-64 PET/CT, Siemens. Images were acquired sixty minutes after tracer injection. CT scanner: Somatom Sensation 16 or 64, Siemens. | All patients were treated with nCRT followed by surgery | AUC=0.78 (Training) AUC=0.74 (Validation) |  |
|  | Wakatsuki et al., 2017 | Enhanced CT: dual-source CT scanner (SOMATOM) | All patients were treated with nCRT followed by surgery | AUC=0.73, P=0.009 |  |
|  | Hou et al., 2017 | Enhanced CT: Philips Brilliance 6 | All patients were treated with deﬁnitive CCRT | ANN: accuracy=0.972, AUC=0.927 (Training) accuracy=0.917, AUC=0.800(Testing)  SVM: accuracy=0.891, AUC=0.818(Training)  accuracy=0.667, AUC=0.600(Testing) |  |
|  | Yip et al., 2016 | PET scanner: GE Discovery or Siemen Biograph (Siemens)  Images were acquired 65 minutes after tracer injection | All patients were treated with nCRT followed by surgery | AUC = 0.72‒0.78 |  |
|  | Rossum et al., 2016 | PET/CT system (Discovery RX, ST, STE, or HR; GE)  Images were acquired 60-90 minutes after tracer injection. Enhanced CT | All patients were treated with nCRT followed by surgery | c-index=0.82(95% CI 0.75–0.88) (apparent)  c-index=0.77 (95% CI 0.70–0.83) (corrected) |  |
|  | Ypsilantis et al., 2015 | Scanner: n.r. | All patients were treated with nCRT | Sensitivity :80.7±11.5Speciﬁcity: 81.6±9.2 Accuracy: 73.4±5.3 |  |
|  | Yip et al., 2014 | Contrast CT: 16–, 128–, or 256–detector row CT scanner (Phillips) | All patients were treated with definitive CCRT | AUC=0.802 |  |
|  | Zhang et al., 2014 | Same as Tan et al., 2013 | All patients were treated with nCRT followed by surgery | AUC=1 (no misclassifications) |  |
|  | Tan et al., 2013 | 16-slice Gemini PET/CT scanner (Philips)  Images were acquired sixty minutes after tracer injection | All patients were treated with nCRT followed by surgery | Texture feature: AUC=0.83, p=0.01  Bin-to-bin and cross-bin histogram distances: AUC=0.78-0.89, p=0.04 |  |
|  | Hatt et al., 2013 | Philips GEMINI PET/CT scanner  Images were acquired sixty minutes after tracer injection | All patients treated with exclusive CCRT | (best) AUC=0.90 |  |
|  | Tan et al., 2013 | Same as Tan et al., 2013 | All patients were treated with nCRT followed by surgery | (best) AUC=0.85 |  |
|  | Tixier et al., 2011 | Gemini PET/CT scanner (Philips).  Images were acquired on average 54 min after injection | All patients were treated with exclusive CCRT | Sensitivity: 76%-92%  Specificity: 56%-91% |  |

Abbreviations used in the table - nCRT: neoadjuvant chemoradiotherapy; CCRT: concurrent chemoradiotherapy.

**Supplementary Table 3: Assessment of methodological quality of included studies**

| **Number** | **Reference** | **Prospective registration** | **Imaging protocol** | **Image pre-processing** | **Segmentation method** | **Repeatability, reproducibility, and dimensionality reduction** | **Correlations with non-handcrafted biomarkers** | **Justification of risk groupings** | **Validation method** | **Comparison to clinical/non-imaging model or Holistic model** | **Discrimination statistics** | **Model calibration** | **Estimation of clinical** **utility** | **External validation availability** |
| --- | --- | --- | --- | --- | --- | --- | --- | --- | --- | --- | --- | --- | --- | --- |
|  | Xie *et al.*, 2021 | **Poor** | **Good**: Several scanners from two institutions, main details provided in suppl | **Poor**: Only resample in suppl | **Moderate**: ROI was manually delineated, but no details about checking | **Good**: Inter-observer, ComBat method for feature harmonization, overlapped genes were used as a filter for the selection of radiomics features (Pearson), univariate analysis, LASSO | **Good**: Correlations with genes and tumor volume | **Moderate**: The cut-off points for the nomograms were determined by Youden Index | **Good**: External validation was performed in a completely independent group of patients, and internal validation | **Good**: Building holistic model combined with genes | **Good**: C-index, AUC, Time-dependent AUC, Kaplan-Meier curve | **Good**: Calibration plots for nomogram models 1 and 2 of training, internal test, external test set | **Good**: Decision curve | **Moderate**: Rad-score based Nomogram is presented, without the coefficients of the features. |
|  | Beukinga *et al.*, 2021 | **Poor** | **Poor**: One scanner, only details of PET acquisition, but no details of CT imaging protocol | **Moderate**: Only resampled and normalized | **Good**: Delineated manually after reaching consensus between 3 collaborating researchers | **Moderate**: No check of either repeatability or reproducibility; univariable logistic regression analysis and LASSO to select features | **Poor**: No | **Poor**: No details about cut-off | **Moderate**: Lacking an independent test or validation cohort; internally validated by a bootstrap approach with 20000 repetitions | **Good**: Building holistic model combined with clinical factors, HER2 and CD44 | **Poor**: Only AUC | **Good**: Calibration plots | **Poor**: No | **Moderate**: The features without coefficients are given. No Nomogram or online code or model. |
|  | Hu *et al.*, 2021 | **Poor** | **Good**: Two scanners, details provided in supplementary table | **Good**: details including resampling, filter, and reconstruction | **Good**: Two senior radiologists respectively delineated the ROI | **Good**: Multi-user delineations to choose features with reproducibility; Pearson correlation coefficient, decision tree, and wrapper method to find features | **Good**: The association of radiomics features and corresponding pathophysiological features (Radiogenomics) | **Moderate**: Cut-offs were determined by Youden index, diagnostic possibilities calculated by cut-off were performed in suppl. | **Good**: External validation was performed in a completely independent group of patients (Guangzhou and Hong Kong) | **Good**: Compared with the clinical model; to elucidate the pathophysiological association with the radiomics signatures (radiogenomics) | **Good**: AUC (95%CI), accuracy, sensitivity, specificity, positive predictive value, negative predictive value; No details of cross-validation or bootstrapping | **Good**: Calibration curve in suppl. But no slope, intercept, P-value was reported. | **Good**: Decision curve in suppl. | **Good**: Pretrained model and selected handcrafted features are presented. Model available online. |
|  | Wang *et al.*, 2021 | **Poor** | **Good**: Scanners from two institutions, main details provided in the article | **Poor**: No details on digital filters or resampling, intensity discretization reported. | **Good**: semiautomatic segmentation method; veriﬁed by a senior radiologist | **Good**: 2-months interval from two-time point to calculate features, intra-class correlation coefficient to collected reproducible features; LASSO | **Poor**: No | **Moderate**: cut-off values of  parameters were determined using receiver operating characteristics (ROC) curve | **Good**: External validation was performed in a completely independent group of patients | **Good**: Building holistic model combined with clinicopathological, dosimetric, and hematological indicators | **Good**: C-index, risk classification | **Good**: Calibration plots for nomogram model | **Good**: Decision curve | **Good**: Nomogram and coefficients of features are provided. |
|  | Li Yimin *et al.*, 2020 | **Poor**： No prospective registration | **Good**: Two scanners, details provided in the article | **Good**: details including resampling, filter, and reconstruction | **Moderate**: Semi-automatic delineation, no details of how many people checked ROI | **Moderate**: No check of either repeatability or reproducibility of features; features selected by repeated Lasso-Cox regression 100 times | **Good**: tested against MTV | **Moderate**: Cut-offs were shown by Kaplan-Meier estimates | **Good**: External validation was performed in a completely independent group of patients (Xiamen and Berlin) | **Good**: Compared with MTV and SUV model in suppl. (failed to identify patients with high or low risk for local recurrence) | **Poor**: Internal and external validated by Kaplan-Meier estimates | **Poor**: No calibration | **Poor**: No decision curve or cost-benefit analysis | **Good**: The selected features and weightings are given. |
|  | Xie *et al.*, 2020 | **Poor** | **Moderate**: Only one scanner, main details provided in reference | **Moderate**: Four filters with different widths; but no details of resampling and intensity discretization | **Good**: The delineation was performed manually with consensus between three radiation oncologists, and refined by an additional thresholding procedure that excluded pixels less than -50HU | **Poor**: No check of either repeatability or reproducibility of features; Cox proportional hazards model were performed on each texture parameter | **Poor**: no correlation testing against non-radiomics features. | **Good**: Median as cut-offs, which were presented in the Figures | **Poor**: Lacking an independent test or validation cohort. | **Poor**: No | **Good**: AUC, P-value; Kaplan-Meier curve | **Poor**: No | **Poor**: No | **Good**: The selected features and coefficients are provided. |
|  | Hu *et al.*, 2020 | **Poor** | **Good**: Two scanners, details provided in supplementary table | **Good**: details including resampling, filter, and reconstruction | **Good**: Two senior radiologists respectively delineated the ROI | **Good**: Multi-user delineations to choose features with reproducibility; Pearson correlation coefficient, decision tree, and wrapper method to find features | **Good**: The association of radiomics features and corresponding pathophysiological features (Radiogenomics) | **Moderate**: Cut-offs were determined by Youden index, diagnostic possibilities calculated by cut-off were performed in suppl. | **Good**: External validation was performed in a completely independent group of patients (Guangzhou and Hong Kong) | **Good**: Compared with the clinical model; to elucidate the pathophysiological association with the radiomics signatures (radiogenomics) | **Good**: AUC (95%CI), accuracy, sensitivity, specificity, positive predictive value, negative predictive value; No details of cross-validation or bootstrapping | **Good**: Calibration curve in suppl. But no slope, intercept, P-value was reported. | **Good**: Decision curve in suppl. | **Moderate**: Only selected features are given, no coefficients. |
|  | Luo *et al.*, 2020 | **Poor** | **Moderate**: Only one scanner, details provided in the article | **Poor**: Only resample information | **Moderate**: Details of segmentation are in the article. The same observer after two months later repeated the tumor segmentation to evaluate the reproducibility | **Good**: Intra-observer and inter-observer intra-class correlation coefficient analysis; LASSO logistic regression to identify the optimal features | **Poor**: No | **Poor**: No details about cut-off | **Poor**: 66 cases were allocated into the testing set from the same institution | **Good**: Building holistic model combined with clinical factors | **Moderate**: AUC, 95%CI | **Poor**: No | **Good**: Decision curve | **Good**: Rad-score-based Nomogram is provided, with coefficients of features. |
|  | Li Yue *et al.*, 2020 | **Poor** | **Good**: Several scanners from one institution, main details provided in suppl | **Poor**: only reconstruction | **Good**: ROI was manually  Delineated by two expert radiation oncologists | **Good**: Inter-observer, test-retest; univariate logistic regression and LASSO | **Moderate**: None  of the selected radiomics feature is correlated with the analyzed clinical features | Poor: No details about cut-off | **Moderate**: Lacking an independent test or validation cohort; internally validated by a bootstrap approach with 2000 repetitions | **Good**: Building holistic model combined with clinical factors | **Poor**: Only AUC | **Good**: Calibration plots | **Good**: Decision curve | **Good**: Rad-score-based Nomogram is provided, with coefficients of features. |
|  | Zhang *et al.*, 2020 | **Poor** | **Good**: Five scanners, details provided in suppl. | **Moderate**: Details of resampling and reconstruction, but no details of filter and intensity discretization | **Moderate**: GTV for radiotherapy planning were used as ROI, but no details of the checking | **Moderate**: No check of repeatability; Pearson correlation was used; Recursive Feature Elimination (RFE) and LASSO was applied to select optimal predictors | **Poor**: no correlation testing against non-radiomics features. | **Poor**: no risk group analysis. | **Good**: External validation was performed in a completely independent group of patients | **Good**: Combined and compared with models developed with clinical variables | **Good**: AUC, accuracy, sensitivity, specificity, positive predictive  value (PPV) and negative predictive value (NPV); 2000 stratified bootstrap | **Good**: Calibration plots for three models | **Poor**: No | **Moderate**: The selected features are provided. No details of calculating Rad-score. |
|  | Du *et al.*, 2020 | **Poor** | **Moderate**: Only one scanner, details provided in the article | **Poor**: No details on digital filters or resampling, intensity discretization reported. | **Good**: semiautomatic segmentation method; veriﬁed by a senior radiologist | **Good**: Spearman rank correlation test to delete the features with correlations greater than 0.9; univariate analysis and LASSO feature selection | **Poor**: No | **Poor**: No details about cut-off | **Moderate**: Lacking an independent test; 50 iterations of 10-fold nested cross-validation, random sampling was conducted | **Good**: Building holistic model combined with clinical features and dosimetric parameters | **Good**: AUC (95%CI), accuracy, sensitivity, specificity, 50 iterations of 10-fold nested cross-validation | **Good**: Calibration plots for nomogram model | **Good**: Decision curve | **Good**: Rad-score-based Nomogram is provided, with coefficients of features. |
|  | Foley *et al.*, 2019 | **Poor** | **Good**: Five scanners, details provided in suppl. | **Moderate**: Derails of STAGE set, but no details of CROSS set. | **Moderate**: Details of segmentation are in the article. But no details of checking | **Moderate**: No check of either repeatability or reproducibility of features; Features from previous knowledge | **Poor**: no correlation testing against non-radiomics features. | **Good**: Patients were separated into quartiles | **Good**: External validation was performed in a completely independent group of patients | **Good**: Compared with the model developed with clinical factors | **Poor**: No details about discrimination metrics | **Good**: Calibration slope and P-value | **Poor**: No decision curve or cost-benefit analysis and no detailed instructions on how to use prognostic model | **Good**: The selected features with coefficients are provided. |
|  | Xie *et al.*, 2019 | **Poor** | **Good**: Two scanners, details provided in the article | **Moderate**: Details other than filters are in the article | **Moderate**: For the training set, GTV for radiotherapy planning were used as ROI, but no details of the checking; For the testing set, details provided in the article | **Moderate**: Pearson correlation method; LASSO to select features; lack inter-observer test or test-retest | **Good**: Spearman rank correlation test was used to assess the correlation of radiomics features with clinical factors and CNAs | **Moderate**: Youden index was used to select the cut-off value | **Good**: External validation was performed in a completely independent group of patients | **Good**: compared with models based on conventional prognostic factors, clinical stage, and ECOG PS | **Good**: C-index, ROC, Kaplan-Meier, and Log-rank | **Poor**: No | **Poor**: No decision curve or cost-benefit analysis and no detailed instructions on how to use prognostic model | **Good**: Rad-score-based Nomogram is provided, with coefficients of features. |
|  | Wang *et al.*, 2019 | **Poor** | **Good**: Several scanners from four institutions, main details provided in suppl | **Poor**: No details on digital filters or resampling, intensity discretization reported. | **Good**: Delineations were performed by three radiation oncologists in each center | **Good**: Stability and intro-observer were tested in suppl; only one feature, uni/multivariate analysis were used | **Good**: Correlations with tumor length or TNM staging | **Moderate**: Cut-off was identified using x-tile software | **Good**: External validation was performed in three completely independent groups of patients | **Good**: Compared with models based on clinical factors | **Good**: C-index, 95%CI; Cox models were used to test the risk model based on compactness(feature) was able to independently predict OS and PFS | **Poor**: No | **Poor**: No | **Good**: Features and cut-off points. |
|  | Chen *et al.*, 2019 | **Poor** | **Moderate**: Only one scanner, main details provided in the article | **Poor**: Only reconstruction information | **Poor**: Delineation based on images and SUV (cut-off value 2.5), but no description of how many doctors checked ROI | **Poor**: Four radiomics features, no check of repeatability, reproducibility, or dimensionality reduction of features | **Poor**: no correlation testing against non-radiomics features. | **Moderate**: Youden index was used to select the cut-off value that was used to develop a scoring system for predicting OS. | **Poor**: Only 16 cases were allocated into the testing set from the same institution | **Poor**: No | **Poor**: Only univariate and multivariate analyses of factors associated with outcomes (HR and P-value) | **Poor**: No | **Poor**: No | **Good**: The details of calculating the risk score are provided. |
|  | Yan *et al.*, 2019 | **Poor** | **Poor**: One scanner, no details on pixel spacing | **Poor**: No details on digital filters or resampling, intensity discretization reported. | **Poor**: No details on segmentation method or indication of how many doctors to check ROI | **Poor**: No check of either repeatability or reproducibility of features; no method of reduction of features | **Poor**: no correlation testing against non-radiomics features. | **Good**: The median change of only one feature as cut-off, two cohorts divided by cut-off were analyzed by KM analysis (P-value) | **Poor**: Lacking an independent test or validation cohort. | **Poor**: No | **Poor**: Only correlation between outcomes and features (r and P-value) | **Poor**: No | **Poor**: No | **Good**: Features are provided, and the cut-off point of one feature is given. |
|  | Yang *et al.*, 2019 | **Poor** | **Moderate**: Only one scanner, main details provided in the article | **Poor**: Details of filters are in the article. No details on intensity discretization were reported. No resample of the voxel size was used. | **Moderate**: Details of segmentation are in the article. But no details of checking | **Moderate**: No check of either repeatability or reproducibility of features; LASSO with 10-fold was applied to select optimal predictors | **Poor**: no correlation testing against non-radiomics features. | **Poor**: no risk group analysis. | **Poor**: Only 11 cases were allocated into the testing set from the same institution | **Poor**: No | **Moderate**: AUC, 95%CI | **Poor**: No | **Poor**: No | **Good**: The selected features with coefficients are provided. |
|  | Jin *et al.*, 2019 | **Poor** | **Moderate**: Only one scanner, main details provided in the article | **Poor**: No details on digital filters or resampling, intensity discretization reported. | **Moderate**: GTV for radiotherapy planning were used as ROI, but no details of the checking | **Poor**: No check of either repeatability or reproducibility of features; PCA was applied before training classifier | **Poor**: no correlation testing against non-radiomics features. | **Poor**: no risk group analysis. | **Moderate**: Only 24 cases were allocated into the testing set from the same institution; randomly partition was performed ten times | **Good**: Building model combined with dosimetric parameters | **Moderate**: Accuracy, AUC; the partition was performed ten times, 10-fold cross-validation was performed on the training set | **Poor**: No | **Poor**: No | **Moderate**: Only selected features are given, no coefficients. |
|  | Foley *et al.*, 2018 | **Poor** | **Moderate**: Only one scanner, main details provided in the article | **Moderate** Details other than filters are in the article | **Moderate**: Details of segmentation are in the article. But no details of checking | **Moderate**: No check of either repeatability or reproducibility of features; Features from previous knowledge | **Poor**: no correlation testing against non-radiomics features. | **Good**: Patients were separated into quartiles | **Moderate**: 101 samples set acquired from the same institution, but different times were allocated into the internal validation set and the model was validated using KM plots. | **Good**: Compared with clinical factors, SUV and MTV and build a holistic model | **Poor**: Log-rank test evaluated significant differences in OS | **Poor**: No | **Poor**: No decision curve or cost-benefit analysis and no detailed instructions on how to use prognostic model | **Good**: The selected features with coefficients are provided. |
|  | Larue *et al.*, 2018 | **Poor** | **Good**: Five scanners, details provided in suppl. | **Good**: details in the article and suppl. | **Moderate**: GTV for radiotherapy planning were used as ROI, but no details of the checking | **Moderate**: No check of repeatability and reproducibility. Selecting the 40 most important predictors and then these features were used as input for RF model | **Poor**: no correlation testing against non-radiomics features. | **Poor**: No details about cut-off | **Good**: External validation was performed in a completely independent group of patients | **Good**: Compared with the model developed with clinical factors | **Good**: AUC, 95%CI, and Kaplan-Meier curve | **Poor**: No | **Poor**: No decision curve or cost-benefit analysis and no detailed instructions on how to use the prediction model | **Moderate**: Only features are provided without coefficients. No Nomogram or online code or model. |
|  | Beukinga *et al.*, 2018 | **Poor** | **Moderate**: Only one scanner, main details provided in the article | **Good**: details in the article | **Moderate**: ROI was manually delineated by an expert radiation oncologist, but no details about checking of other doctors | **Good**: Details of intra-class correlation coefficient and Akaike Information Criterion. LASSO to select features. | **Poor**: no correlation testing against non-radiomics features. | **Poor**: No details about cut-off | **Moderate**: Lacking an independent test. Only internal validation by bootstrap resampling with 20000 replicates. | **Good**: Compared with clinical factors, and SUV and build a holistic model | **Good**: ROC and discrimination slope | **Good**: Calibration slope and intercept | **Poor**: No | **Moderate**: Only features are provided without coefficients. No Nomogram or online code or model. |
|  | Riyahi *et al.*, 2018 | **Poor** | **Moderate**: Only one scanner, main details provided in reference | **Poor**: No details on digital filters or resampling, intensity discretization reported. | **Moderate**: Details of segmentation are in the article. But no details of checking | **Moderate**: Used a pair-wise correlation cutoff, and selected features by SVM coupled with LASSO | **Poor**: no correlation testing against non-radiomics features. | **Poor**: No details about cut-off | **Poor**: Lacking an independent test or validation cohort. | **Good**: Compared and combined with PET/CT features model | **Moderate**: AUC, accuracy, sensitivity, specificity | **Poor**: No | **Poor**: No | **Moderate**: Only features. |
|  | Paul *et al.*, 2017 | **Poor** | **Poor**: Only scanner and only voxel size were provided | **Poor**: No details on digital filters or resampling, intensity discretization reported. | **Poor**: No details on segmentation method or indication of how many doctors to check ROI | **Moderate**: No check of repeatability. Spearman’s rank correlation analysis to eliminate correlated features; Genetic Algorithm based on Random Forest and LASSO to select features | **Poor**: no correlation testing against non-radiomics features. | **Poor**: no risk group analysis. | **Poor**: Lacking an independent test or validation cohort. | **Poor**: No | **Good**: Cross-validation method; AUC (95%CI), accuracy, sensitivity, specificity, positive predictive value, the negative predictive value | **Poor**: No | **Poor**: No | **Moderate**: Only features. |
|  | Desbordes *et al.*, 2017 | **Poor** | **Poor**: Only scanner and no details of scan protocols | **Good**: details in the article | **Moderate**: Details of segmentation are in the article. But no details of checking | **Moderate:** Spearman correlation rank analysis; Random Forest to select features | **Good:** Correlation with SUV and MTV | **Poor**: No details about cut-off | **Moderate**: Lacking an independent test. Only randomly divided the database into training and test set, and repeated 10 times | **Good**: Compared with models based on clinical factors, SUV and MTV | **Good**: AUC, accuracy, sensitivity, specificity; Kaplan-Meier survival curves | **Poor**: No | **Poor**: No | **Moderate**: Only features. |
|  | Nakajo *et al.*, 2017 | **Poor** | **Moderate**: Only one scanner, main details provided in reference | **Moderate**: Reconstruction and resampling of the intensity of FDG uptake represented in the article | **Good**: ROI was manually set, tumor boundaries were then automatically contoured. The focal uptake in the primary lesion was visually interpreted by three doctors | **Poor**: No check of either repeatability or reproducibility of features; univariate and multivariate analysis of features | **Good:** Correlation with PET features | **Moderate**: Cut-offs by using ROC, presented in TableS2; K-M curves of different patients divided by cut-off | **Poor**: Lacking an independent test or validation cohort. | **Good**: compared with models based on clinical factors | **Good**: AUC, accuracy, sensitivity, specificity; Kaplan-Meier survival curves | **Poor**: No | **Poor**: No | **Moderate**: Only features. |
|  | Beukinga *et al.*, 2017 | **Poor** | **Moderate**: Only one scanner, main details provided in the article | **Poor**: No details on digital filters or resampling, intensity discretization reported. | **Good**: The delineation was performed manually with consensus between three radiation oncologists | **Good**: The stability of features was evaluated with the intra-class correlation (ICC); potential predictors that met the Akaike Information Criterion (AIC) were considered significant and selected by LASSO | **Poor**: no correlation testing against non-radiomics features. | **Poor**: No details about cut-off | **Moderate**: Lacking an independent test or validation cohort; internally validated by a bootstrap approach with 2000 repetitions | **Good**: Compared with the model developed with clinical factors | **Good**: AUC and discrimination slope; internally validated by bootstrap with 2000 repetitions | **Good**: Calibration slope and intercept | **Poor**: No | **Poor**: No exact features and coefficients or final model are given. |
|  | Wakatsuki *et al.*, 2017 | **Poor** | **Moderate**: Only one scanner, main details provided in the article | **Poor**: No details on digital filters or resampling, intensity discretization reported. | **Poor**: No details about segmentation method and checking | **Poor**: No check of either repeatability or reproducibility; CT number of a primary tumor as a feature, no need to do dimensionality reduction | **Good**: Correlation with Ki-67, P53, and CK5/6 expression | **Moderate**: According ROC to define cut-off, and used it to calculate Kaplan-Meier | **Poor**: Lacking an independent test or validation cohort. | **Poor**: No | **Moderate**: AUC and Kaplan-Meier | **Poor**: No | **Poor**: No | **Good**: Cut-off point of the feature. |
|  | Hou *et al.*, 2017 | **Poor** | **Moderate**: Only one scanner, details provided in the article | **Moderate**: Only resampled and normalized | **Good**: Manually delineated by two doctors, and reviewed by the third doctor | **Moderate**: Intra-class correlation coefficient was used to quantify reproducibility; Kruskal-Wallis test and wrapper-based feature selection method to select features | **Poor**: No | **Moderate**: According to ROC to define cut-off | **Poor**: Only 12 cases were allocated into the testing set from the same institution | **Poor**: No | **Good**: AUC, accuracy, sensitivity, specificity, positive predictive value, negative predictive value; No details of cross-validation or bootstrapping | **Poor**: No | **Poor**: No | **Moderate**: Only features are provided without coefficients. No Nomogram or online code or model. |
|  | Yip *et al.*, 2016 | **Poor** | **Poor**: Two scanners, but no details of imaging protocol | **Poor**: Only reconstruction of PET data | **Poor**: No details about segmentation method and checking | **Moderate**: Three features were chosen due to a previous report predicting potential | **Good**: Correlation with MTV and tumor volume | **Poor**: No details about cut-off | **Poor**: Lacking an independent test or validation cohort. | **Moderate**: Only AUC of MTV was reported, but no figures | **Poor**: AUC of models based on different algorithm propagated contours | **Poor**: No | **Poor**: No | **Moderate**: Only features. |
|  | Rossum *et al.*, 2016 | **Poor** | **Good**: Several scanners, main details provided in suppl | **Good**: Details were provided in suppl | **Moderate**: Semiautomatic delineation method, followed by 1 interpreter | **Good**: Test-retest (ICC), Spearman rank correlation; univariable and multivariable analysis to select features; all these details provided in suppl | **Poor**: No | **Poor**: No details about cut-off | **Moderate**: Lacking an external test. Only internal validation by bootstrap method with 1000 repetitions | **Good**: Compared with models based on clinical factors, and different PET features | **Poor**: Apparent and corrected C-indices | **Good**: Calibration plot for all four models | **Good**: Decision-curve | **Good**: The selected features with coefficients are provided. |
